# Nonlinear Vortex-Induced Vibrations of Fluid-Conveying Pipes with Gravity-Induced Slight Initial Curvature

**DOI:** 10.3390/ma19163426

**Published:** 2026-08-12

**Authors:** Bin Zhang, Hui-Feng Wang, Zhen-Zhong Hu, Hui Wang, Zi-Qiang Ni, Sun-Wei Li

**Affiliations:** 1 Offshore Oil Engineering Co., Ltd., Tianjin 300461, China; zhangbin77@cnooc.com.cn (B.Z.); wanghf22@cnooc.com.cn (H.-F.W.); wanghui33@cnooc.com.cn (H.W.); nizq@cnooc.com.cn (Z.-Q.N.); 2 Shenzhen International Graduate School, Tsinghua University, Shenzhen 518055, China; huzhenzhong@tsinghua.edu.cn

**Keywords:** vortex-induced vibration, gravity-induced slight initial curvature, fluid-conveying pipes, van der Pol equation, numerical calculations

## Abstract

**Highlights:**

- A vortex-induced vibration prediction model for slightly curved pipes is developed.
- Gravity-induced static curvature reduces the structural response mode.
- Tension and internal flow velocity affect the static curvature of the pipe.

**Abstract:**

Vortex-induced vibration (VIV) is one of the main causes of fatigue failure in subsea pipelines and has recently attracted significant attention from researchers. Previous studies have mainly focused on idealized straight pipes, with limited consideration of gravity-induced slight curvature in free-spanning fluid-conveying pipes. In reality, the deformation configuration of a free-spanning fluid-conveying pipe is not fixed but varies with parameters such as internal flow velocity and tension, which in turn affect its dynamic behavior. A theoretical model, taking into account the axial stretching effect and the gravity-induced initial slight curvature, is developed to predict the VIV responses of free-spanning fluid-conveying pipes. The governing equations are derived based on Hamilton’s principle. The interaction between the external flow and the pipe structure is simulated using the van der Pol equation. By combining the Galerkin method and the Runge–Kutta method, the vibration responses of the pipe are obtained. The accuracy of the proposed model is validated by comparing the predicted VIV response curves and bifurcation diagrams with those reported in previous studies. The initial static deformation of the structure under different tensions and internal velocities is obtained through numerical calculations. It is found that the gravity-induced slight curvature leads to a reduction in the VIV response mode. The static deformation of the pipe decreases with increasing axial tension, while it increases with increasing internal flow velocity. Under the same external flow velocity, the gravity-induced initial deformation reduces the dominant vibration frequency and causes the vibration response to transition from quasi-periodic to periodic motion.

## 1. Introduction

The fluid-conveying pipe, as a core component in industrial production, is widely used in fields such as aerospace, ocean engineering, nuclear energy, civil engineering, and chemical engineering [1,2,3]. Considering that the pipe transports fluids that are essential to the operation of the system, its failure can lead to system shutdown and result in significant economic losses. The failure modes of pipe systems are diverse and complex, even though the structure itself is simple. Taking submarine pipelines as an example, when transporting high-temperature/high-pressure oil and gas, the pipe structure undergoes thermal expansion, which in turn induces various failure modes, such as upward buckling in partially buried pipes [4], lateral buckling in unburied pipes [5], and axial walking in pipes subjected to cycles of temperature and pressure variation [6,7]. In addition, pipes supported at both ends may experience buckling when the internal flow velocity exceeds the critical value [8]. The cantilevered pipes, on the other hand, are more prone to flutter problems [9]. Moreover, variations in axial tension can lead to parametric resonance [10]. The most significant problem that a riser (vertical pipe) or a submarine pipe (horizontal pipe) faces may be vortex-induced vibrations (VIV), where the external flow is perpendicular to the pipe axis [11]. Consequently, the mechanical properties and failure modes of fluid-conveying pipes have remained a key concern for academic researchers, and the findings provide valuable references for the engineering design of pipes [12,13,14].

When external flow passes through a pipeline, viscous forces cause the fluid in the boundary layer to separate, and vortices to shed in an alternating manner behind the structure. The alternating shedding of vortices exerts fluctuating pressures on the pipe, which induces VIV [15,16]. When VIV occurs, the pipe experiences small-amplitude, high-frequency vibrations, which exacerbate its fatigue condition. In fact, VIV has been recognized as a major contributor to fatigue in submarine pipelines [17], accounting for approximately 21% of incidents [18]. When the vortex shedding frequency synchronizes with the structural vibration frequency, lock-in resonance occurs, causing a significant increase in the pipe vibration amplitude and making the pipe more susceptible to fatigue failure [19]. Given the importance of the VIV, a considerable number of previous studies have focused on understanding the VIV characteristics under different external conditions. Experiments [20,21], numerical simulations [22,23], and semi-empirical models [24,25] are the main approaches used to analyze VIV. Although the VIV responses predicted through experiments are generally closer to real-world conditions, some key flow parameters, such as spatial variation in flow velocities, are often difficult to control precisely in the laboratory. Additionally, the facility requirements and costs associated with experiments limit their practical use in predicting the behavior of a pipe undergoing VIV. Numerical simulations, on the other hand, suffer from long computation times and insufficient realism/reliability when the flow is at the high Reynolds number state. Semi-empirical methods have attracted significant attention due to their computational efficiency and clear physical interpretation. Specifically, the wake oscillator model has been widely applied [26,27,28] to discern the VIV of both rigid and flexible pipes. The wake oscillator model does not directly simulate the flow field but instead establishes the connections among the key features of VIV observed experimentally. Bishop and Hassan [29] proposed the general form of the wake oscillator model, using the van der Pol oscillator equation to describe the fluctuating pressures on the structure. Facchinetti et al. [30] followed the trend to investigate three coupling schemes of the van der Pol equation (displacement, velocity, and acceleration) and found that the acceleration-coupled scheme provided good predictions of the fundamental VIV response characteristics. Consequently, the van der Pol equation with the acceleration coupling scheme has been widely adopted for predicting VIV in pipes [31,32,33]. Recently, modifications have been suggested based on variants of the wake oscillator to achieve higher prediction accuracy by improving structural models [34] or modifying the van der Pol equation [35].

Using the wake oscillator model, a number of factors are found to be influential on the dynamic characteristics of the VIV. First, it is found that the tension variations in the pipe, due to factors such as thermal loads [36,37,38] and platform motion [39,40], lead to changes in the dynamic characteristics of the pipe structure [41], affecting its stability [42], and reducing its fatigue life span [43]. Zhao et al. [44] investigated the VIV responses of pipe-in-pipe systems based on the wake oscillator model and the Euler-Bernoulli beam model. Their research revealed that the increase in tension leads to a decrease in the VIV responses of the inner and outer pipes, while the frequency ratio and the collision time between the inner and outer pipes increase with the increasing tension. Second, it is found that the internal flow velocity in a fluid-conveying pipe is a key factor in determining the dynamic characteristics, which may introduce Coriolis force and centrifugal force on the pipe [45]. Research has shown that the variation in internal flow velocity can induce parametric resonance in the pipe [46]. Furthermore, when the internal flow velocity exceeds a certain value, the VIV behavior of the pipe undergoes significant changes [9,46].

**Figure 1:**
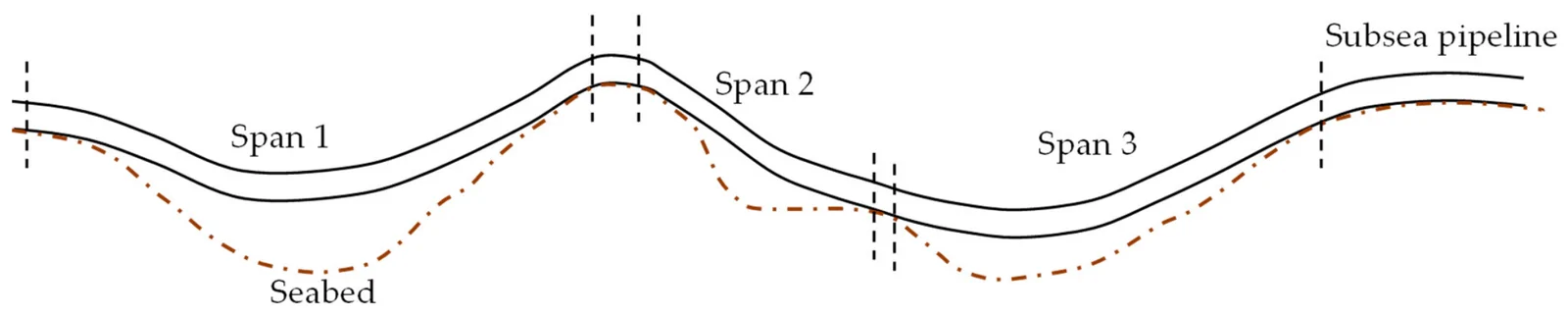
Diagram of a subsea pipeline laid on the seabed. Solid line: subsea pipeline; dashed line: span ends; dash-dotted line: seabed.

It is noted that previous studies on VIVs of fluid-conveying pipes have mostly focused on straight structures. Due to factors such as manufacturing errors, installation inaccuracies, and gravity, pipes in practice, however, are typically slightly curved [47]. As shown in Figure 1, for example, at the free-span location, the pipeline laid on the seabed naturally deflects downward under gravity. Therefore, it is necessary to consider the gravity-induced initial slight curvature when investigating the VIV characteristics of the subsea fluid-conveying pipes. Recently, there have been studies focusing on the dynamic characteristics of pipes with initial geometric imperfections [48,49], and have found that even small initial curvatures can significantly impact the dynamic behavior of the pipe [50]. Ye et al. [51] studied the dynamic behavior of slightly curved pipes conveying supercritical fluid and found that initial curvature significantly affects the structure’s natural frequency and critical internal flow velocity. Shao and Ding [52] investigated the nonlinear vibration characteristics of fluid-conveying pipes with elastic support, considering the initial static deformation caused by gravity. Their research indicates that gravity significantly influences the structure’s lower-order natural frequencies. However, all the above studies did not consider the effect of VIV.

More recently, Xu et al. [53] studied the VIV of slightly curved pipes in oscillatory flow and found that the effect of initial deformation on the in-line response is greater than its effect on the cross-flow response. However, this study assumed that the initial deformation follows a fixed sinusoidal function, which oversimplifies the realistic condition. In reality, the static deformation of the pipe may result from the combination of internal flow velocity, tension and gravity, and hence not follow the fixed sinusoidal shape. In general, investigations on the dynamic characteristics of slightly curved fluid-conveying pipes undergoing VIVs are still limited.

The present study aims to investigate the VIV behavior of the fluid-conveying pipes with gravity-induced initial slight curvature under the condition of varying key factors such as the internal fluid velocity and the tension. Previous studies have mostly assumed a fixed initial deflection shape [53,54], and the initial imperfection is generally represented by a sinusoidal function. This assumption may lead to the following limitations. First, the assumption of a fixed sinusoidal imperfection primarily affects the lower-order natural frequencies, especially the first mode, while having a relatively small influence on the higher-order natural frequencies [52,53]. Second, variations in tension and internal flow velocity can influence the static configuration of the pipe, which in turn affects the structural stiffness and the corresponding dynamic behavior. However, this coupled effect has not been considered in existing studies. The present study attempts to accurately calculate the realistic initial deformation due to gravity, which varies with the internal flow velocity and the applied tension. The conclusions of the study are expected to provide guidance for the design of subsea free-span pipelines.

The manuscript is organized as follows: In Section 2, a VIV prediction model for the fluid-conveying pipes considering gravity and axial coupling is established. In Section 3, the performance and reliability of the proposed model are verified through a comparison with existing calculation results. In Section 4, the differences in the VIV behavior, with and without the consideration of the gravity effect, are analyzed. Finally, the main conclusions of the present study are summarized in Section 5.

## 2. Development of the Theoretical Model

**Figure 2:**
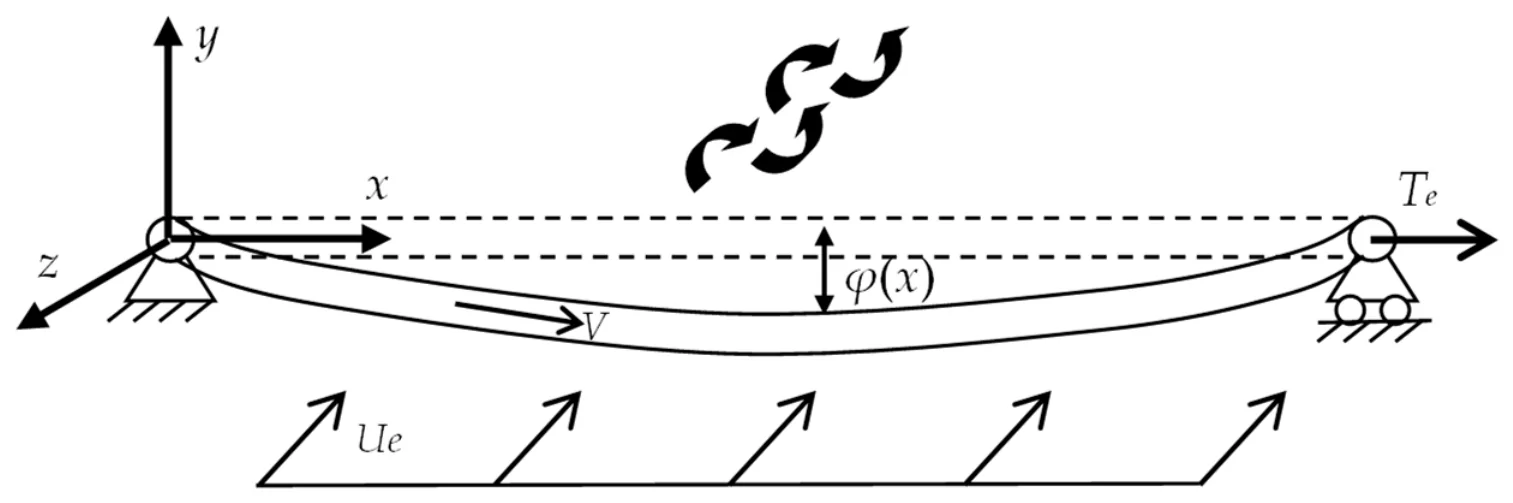
Diagram of a fluid-conveying pipe subjected to external flow.

Figure 2 shows the schematic diagram of a horizontally fluid-conveying pipe, which is the problem targeted in the present study. Similar to previous studies [9,25], the boundary conditions of the subsea free-span pipeline are assumed to be simply supported at both ends. This assumption is reasonable for two main reasons. On the one hand, the pipe-soil interaction is highly complex, and its mechanical behavior depends on factors such as soil stiffness, embedment depth, and local scour, which are difficult to characterize in practice. On the other hand, the seabed soil can restrain transverse displacement through bearing and embedment; however, local soil yielding, gap formation, and cyclic unloading under hydrodynamic loading reduce the rotational stiffness at the pipe–soil interface. Therefore, the simply supported boundary condition has been widely adopted [9,25]. This simplification can provide high computational efficiency and capture the main vibration characteristics of the pipe. It should be noted that the actual pipe–soil interaction may provide finite translational and rotational stiffness at the pipe ends. Variations in these stiffness values can affect the structural constraint conditions and consequently modify the natural frequencies and VIV responses. Since this study mainly focuses on the effect of gravity-induced initial curvature on VIV behaviors, the simply supported boundary condition is adopted to avoid the additional uncertainties associated with complex soil–pipe interactions.

The internal and external flow velocities of the pipe are denoted as *V* and *U_e_*, respectively. A Cartesian coordinate system (*x*, *y*, *z*) is established to describe the motion state of the pipe. The origin of the coordinate system is located at the left endpoint of the structure. The *z*-axis lies along the external flow plane, the *y*-axis is perpendicular to the external flow plane, and the *x*-axis goes through the pipe. Due to the gravitational force, the axis of the suspended pipeline undergoes initial static deformation, and the deflection along the pipe is denoted by *φ*(*x*). The fluid-conveying pipe is modeled as an Euler–Bernoulli beam due to its large aspect ratio.

### 2.1. Dynamic Model

Previous studies have demonstrated that, when lock-in occurs, the vibration amplitude of the VIV in the *y*- direction is significantly larger than the amplitude in the *x* and *z*- directions [34]. Therefore, the following analysis will primarily focus on the VIV response of the pipe in the *y*-direction, i.e., the transverse direction. In this study, the dynamic equation of the straight pipe is first established. Then, the dynamic equation of the slightly curved pipe is developed based on the straight pipe model.

The dynamic governing equations of the straight pipe subjected to external flow are derived using Hamilton’s principle, given by [54]

(1) $$\delta \int_{t_{1}}^{t_{2}}\left(T_{total}-V_{total}\right)dt+\delta \int_{t_{1}}^{t_{2}}Wdt=0,$$

where *δ*(·) is the variational operator, *t* denotes time, the terms *T_total_*, *V_total_* and *W* correspond to the system’s total kinetic energy, total potential energy and the work done by external force, respectively. The system’s total kinetic energy *T_total_* comprises the kinetic energy of the pipe and the kinetic energy of the internal flow, which can be expressed as

(2) $$T_{total}=T_{p}+T_{f}=\frac{m_{p}}{2}\int_{0}^{L}\left({\dot{u}}^{2}+{\dot{v}}^{2}\right)dx+\frac{m_{f}}{2}\int_{0}^{L}\left\{{\left[\dot{u}+V\left(1+u^{\prime }\right)\right]}^{2}+{\left(\dot{v}+Vv^{\prime }\right)}^{2}\right\}dx,$$

where *u* and *v* represent the vibration amplitude of the pipe in the *x*- and *y*- directions, respectively, *m_p_* and *m_f_* denote the mass per unit length of the pipe and the internal fluid, respectively, and *L* is the length of the pipe. The primes and dots represent partial differentiation with respect to the spatial coordinate *x* and the time *t*, respectively.

According to Li and Yang [54], the strain energy *V_p_* of the slightly curved pipe can be expressed as

(3) $$V_{p}=\frac{EA}{2}\int_{0}^{L}{\left(u^{\prime }+\frac{1}{2}{v^{\prime }}^{2}+\frac{T_{e}}{EA}\right)}^{2}dx+\frac{EI}{2}\int_{0}^{L}{v^{\prime \prime }}^{2}dx,$$

where the term inside the parentheses on the right-hand side denotes the nonlinear stretching strain that accounts for the effect of the initial curvature, *T_e_* represents the tension, *E* is the modulus of elasticity, *I* and *A* denote the moment of inertia and the cross-sectional area of the pipe, respectively.

In addition to the strain energy *V_p_*, the total potential energy *V_total_* should also account for the potential energy *V_g_* caused by the wet weight of the pipe, which was not previously considered [54]. The gravitational potential energy can be calculated as

(4) $$V_{g}=\left(m_{p}+m_{f}-\frac{\rho_{0}\pi D^{2}}{4}\right)g\int_{0}^{L}vdx,$$

where *g* denotes the gravitational acceleration, *ρ*_0_ is the external flow density, and *D* is the outer diameter of the pipe. In Equation (4), *ρ*_0_π*D*^2^*g*/4 represents the buoyancy force acting on the pipe per unit length. The virtual work δ*W* is determined by

(5) $$\delta W=\int_{0}^{L}\left(F_{x}\delta u+F_{y}\delta v\right)dx-c\int_{0}^{L}\left(\dot{u}\delta u+\dot{v}\delta v\right)dx,$$

where *c* is the damping coefficient.

Studies showed that the external flow induces VIV, resulting in pulsating pressure on the pipe. The pulsating pressure is primarily in the plane perpendicular to the pipe axis [34], meaning that the axial forces exerted on the pipe by the external flow are relatively small. Therefore, it is assumed that the hydrodynamic force acting on the pipe in the axial direction is zero, i.e., *F_x_* = 0. The hydrodynamic model from Dai et al. [55] is adopted in the study. According to Dai et al. [55], the transverse hydrodynamic force consists of the damping force and the lift force. Additionally, when the pipe undergoes transverse vibration, it is subjected to the effect of added mass. Therefore, the hydrodynamic forces acting on the pipe can be expressed as follows

(6) $$F_{y}=-m_{a}\ddot{v}+f_{D}+f_{L}$$

where *m_a_* = *C_a_ρ*_0_π*D*^2^/4 represents the inertial force induced by the vibration of the pipe in water. The empirical coefficient *C_a_* is affected by the flow conditions and boundary effects. Previous studies have shown that *C_a_* tends to increase when the pipe is close to the seabed [56,57]. The present study focuses on the influence of gravity-induced slight initial curvature on the VIV responses. Therefore, the seabed-induced modification of *C_a_* is not considered. This assumption is reasonable for cases where the pipe-seabed gap is sufficiently large compared with the pipe diameter. The pipe is assumed to vibrate in the subcritical Reynolds number regime, and *C_a_* = 1 is adopted as a representative value for a circular cylinder based on previous studies [34]. The damping force *f_D_* and the lift force *f_L_* can be expressed as [55]

(7) $$f_{D}=-\frac{1}{2}C_{D}\rho_{0}DU_{e}\dot{v},\;f_{L}=\frac{1}{2}C_{L}\rho_{0}DU_{e}^{2},$$

where *C_D_* is the damping coefficient, and *C_L_* represents the lift coefficient. Facchinetti et al. [30] used the van der Pol equation with structural acceleration coupling terms to describe the variation in the lift coefficient and found that this method could predict the fundamental characteristics of VIV. According to Ref. [30], the lift coefficient can be expressed as

(8) $$C_{L}=C_{L0}\frac{q\left(x,t\right)}{2},$$

where *C_L_*_0_ is an empirical parameter. The values of *C_D_* and *C_L_*_0_ are obtained from forced-oscillation experiments and may vary with changes in the flow conditions during VIV [58,59]. Previous experimental studies have calibrated the values of *C_D_* and *C_L_*_0_ and suggested that, for circular cylinders in the subcritical Reynolds number regime, *C_D_* = 1.2 can be adopted [58], while *C_L_*_0_ = 0.3 is commonly used over a wide range of Reynolds numbers [58,60]. In the present study, the pipe is assumed to vibrate under stable subcritical Reynolds number conditions. Therefore, according to Refs. [30,58,60], *C_D_* and *C_L_*_0_ are set to 1.2 and 0.3, respectively. *q*(*x*, *t*) is the wake variable, which can be determined using the following van der Pol equation.

(9) $$\ddot{q}+\varepsilon_{y}\omega_{s}\left(q^{2}-1\right)\dot{q}+\omega_{s}^{2}q=\frac{A_{y}}{D}\ddot{v},$$

where *ε_y_* and *A_y_* are empirical parameters that primarily affect the predicted response amplitudes. It is noted that a universal criterion for determining these parameters has not yet been established. Previous studies have mainly provided recommended ranges rather than fixed values [8,30]. Facchinetti et al. [30] suggested that *A_y_*_/_*ε_y_* should be within the range of 30–50 and demonstrated that *ε_y_* = 0.3 and *A_y_* = 12 provide reasonable predictions for VIV responses [30,60]. Many subsequent studies have also selected these empirical parameters within the recommended range based on sensitivity analyses [8,34]. In the present study, the pipe is assumed to vibrate in the subcritical Reynolds number regime, which is consistent with the assumptions adopted by Facchinetti et al. [30] and Dai et al. [55]. Therefore, the empirical parameters are set as *ε_y_* = 0.3 and *A_y_* = 12, following the values recommended by these previous studies. *ω_s_* represents the vortex shedding frequency, which is given by

(10) $$\omega_{s}=2\pi S_{t}\frac{U_{e}}{D}.$$

The Strouhal number *S_t_* describes the relationship between the flow velocity, cylinder diameter, and vortex shedding frequency. It may vary with flow conditions, such as the Reynolds number and turbulence intensity [61]. The present study mainly focuses on the influence of gravity-induced slight initial curvature on the VIV responses, while the flow condition is assumed to remain unchanged. Therefore, *S_t_* is treated as a constant parameter in the present model. Previous studies have commonly adopted a constant value of *S_t_* within the range of 0.16–0.2 for circular cylinders [10,34,55]. Zhang et al. [34] conducted a sensitivity analysis of the effect of *S_t_* on the VIV responses. Since the present work is an extension of the model proposed by Dai et al. [55], the same value of *S_t_* = 0.2 is adopted.

It can also be obtained from Equation (10) that the vortex shedding frequency is determined by the external flow velocity and the Strouhal number. In the present model, the vortex shedding frequency is not directly modified by changes in the structural natural frequency caused by gravity effect, axial tension, or internal flow velocity. Instead, these factors affect the structural natural frequency and consequently influence the occurrence of lock-in. When the structural frequency approaches the vortex shedding frequency, synchronization occurs, and the vibration frequency gradually shifts toward the lock-in frequency.

Substituting Equations (2)–(5) into Equation (1) and performing the variational operation yields the dynamic equations for the slightly curved pipe as follows

(11) $$\left(m_{p}+m_{f}\right)\ddot{u}+2m_{f}V{\dot{u}}^{\prime }+m_{f}V^{2}u^{\prime \prime }+c\dot{u}-EA\left(u^{\prime \prime }+v^{\prime }v^{\prime \prime }\right)=0,$$

$$\left(m_{p}+m_{f}\right)\ddot{v}+2m_{f}V{\dot{v}}^{\prime }+m_{f}V^{2}v^{\prime \prime }+c\dot{v}+EIv^{⁗}-T_{e}v^{\prime \prime }$$

(12) $$+\left(m_{f}+m_{p}-\frac{\rho_{0}\pi D^{2}}{4}\right)g-EA\left(\frac{3}{2}{v^{\prime }}^{2}v^{\prime \prime }+u^{\prime }v^{\prime \prime }+u^{\prime \prime }v^{\prime }\right)=F_{y},$$

### 2.2. Model Simplification

Since the dynamic response of the structure in the transverse direction is the major concern, the governing equation of the transverse motion of the structure, which accounts for the axial stretching effect [62], can be derived from Equations (11) and (12). Equation (11) can be simplified through the following steps: first, ignore the time-related terms, which is equivalent to neglecting axial motion. Second, omit the stiffness term *m_f_V*^2^, since it is negligible compared to the term *EA* [46,62]. Consequently, the equation reduces to

(13) $$u^{\prime \prime }=-v^{\prime }v^{\prime \prime }.$$

Integrating Equation (13) yields

(14) $$u=-\int_{0}^{x}\left(\frac{1}{2}{v^{\prime }}^{2}\right)dx+c_{1}x+c_{2},$$

where *c*_1_ and *c*_2_ are the coefficients determined by applying the boundary conditions. Considering the pipe is simply supported as shown in Figure 1, it can be concluded that

(15) $${u|}_{x=0}=0,{u|}_{x=L}=0.$$

Combining Equations (14) and (15), it is obtained that

(16) $$c_{1}=\frac{1}{L}\int_{0}^{L}\left(\frac{1}{2}{v^{\prime }}^{2}\right)dx,\;c_{2}=0$$

Substituting Equation (16) into Equation (14) and combining it with Equation (12), the transverse dynamic equation of the pipe, accounting for axial coupling effects, can be expressed as

$$\left(m_{p}+m_{f}\right)\ddot{v}+2m_{f}V{\dot{v}}^{\prime }+m_{f}V^{2}v^{\prime \prime }+c\dot{v}+EIv^{⁗}$$

(17) $$+\left(m_{f}+m_{p}-\frac{\rho_{0}\pi D^{2}}{4}\right)g-T_{e}v^{\prime \prime }-\frac{EA}{L}v^{\prime \prime }\int_{0}^{L}\left(\frac{1}{2}{v^{\prime }}^{2}\right)dx=F_{y}.$$

To nondimensionalize the governing equations, a set of nondimensional variables is introduced, as listed in Table 1.

Then, the VIV prediction equations for the slightly curved pipe conveying fluid, i.e., Equations (9) and (17), can be rewritten in the following dimensionless form

(18) $$\ddot{\bar{v}}+2u_{i}\sqrt{\beta }{\dot{\bar{v}}}^{\prime }+u_{i}^{2}{\bar{v}}^{\prime \prime }+\left(\sigma_{f}+\sigma_{s}\right)\dot{\bar{v}}+{\bar{v}}^{⁗}+\Gamma -\gamma {\bar{v}}^{\prime \prime }-\chi {\bar{v}}^{\prime \prime }\int_{0}^{1}\left(\frac{1}{2}{{\bar{v}}^{\prime }}^{2}\right)d\xi =\alpha q,$$

(19) $$\ddot{q}+\varepsilon_{y}\Omega_{s}\left(q^{2}-1\right)\dot{q}+\Omega_{s}^{2}q=A_{y}\ddot{\bar{v}},$$

where the primes and dots in Equations (18) and (19) represent partial differentiation with respect to *ξ* and *τ*, respectively.

To establish the dynamic equation of the pipe at the gravity-induced equilibrium position, the solution of Equation (18) is decomposed into a quasi-static displacement $\bar{\varphi }$ = *φ*/*D*, which is affected by the tension and internal flow velocity, and a dynamic component ${\bar{v}}_{d}$, such that

(20) $$\bar{v}\left(\xi ,\tau \right)=\bar{\varphi }\left(u_{i},\gamma ,\xi \right)+{\bar{v}}_{d}\left(\xi ,\tau \right),$$

Substituting Equation (20) into Equation (19) gives

$$\left(\ddot{\bar{\varphi }}+{\ddot{\bar{v}}}_{d}\right)+2u_{i}\sqrt{\beta }\left({\dot{\bar{\varphi }}}^{\prime }+{{\dot{\bar{v}}}_{d}}^{\prime }\right)+u_{i}^{2}\left({\bar{\varphi }}^{\prime \prime }+{{\bar{v}}_{d}}^{\prime \prime }\right)+\left(\sigma_{f}+\sigma_{s}\right)\left(\dot{\bar{\varphi }}+{\dot{\bar{v}}}_{d}\right)+\left({\bar{\varphi }}^{⁗}+{{\bar{v}}_{d}}^{⁗}\right)+\Gamma$$

(21) $$-\gamma \left({\bar{\varphi }}^{\prime \prime }+{{\bar{v}}_{d}}^{\prime \prime }\right)-\chi \left({\bar{\varphi }}^{\prime \prime }+{{\bar{v}}_{d}}^{\prime \prime }\right)\int_{0}^{1}\left(\frac{1}{2}{{\bar{\varphi }}^{\prime }}^{2}+\frac{1}{2}{{{\bar{v}}_{d}}^{\prime }}^{2}+{\bar{\varphi }}^{\prime }{{\bar{v}}_{d}}^{\prime }\right)d\xi =\alpha q.$$

(22) $$\ddot{q}+\varepsilon_{y}\Omega_{s}\left(q^{2}-1\right)\dot{q}+\Omega_{s}^{2}q=A_{y}\left(\ddot{\bar{\varphi }}+{\ddot{\bar{v}}}_{d}\right),$$

The present study suggests numerical methods to solve Equations (21) and (22) given their partial differential nature. Based on the Galerkin method, these equations can be transformed into a set of ordinary differential equations using the following variable separations

(23) $${\bar{v}}_{d}=\sum_{i=1}^{N}\phi_{i}\left(\xi \right){\bar{\bar{v}}}_{d,i}\left(\tau \right),\;q\left(\xi ,\tau \right)=\sum_{i=1}^{N}\phi_{i}\left(\xi \right){\bar{q}}_{i}\left(\tau \right),$$

where $\phi_{i}=\sqrt{2}\sin(i\pi \xi )$ is adopted as the admissible function, which satisfies the simply supported boundary conditions and the following orthogonality condition

(24) $$\int_{0}^{1}\phi_{i}\left(\xi \right)\phi_{j}\left(\xi \right)d\xi =\left\{\begin{array}{l} 0,i\neq j\\ 1,i=j \end{array}\right.,i=1,2\ldots N.$$

It is noted that the adopted functions are based on the eigenfunctions of a simply supported beam and are used as admissible functions rather than the exact eigenfunctions of the initially curved pipe. The effect of the initial curvature is incorporated into the governing equations through the nonlinear strain. For the considered pipe configuration, the gravity-induced initial curvature is relatively small compared with the span length; therefore, the use of straight-beam admissible functions is considered a reasonable approximation. *N* is the truncation order. ${\bar{\bar{v}}}_{i}\left(\tau \right)$ and ${\bar{q}}_{i}\left(\tau \right)$ are the generalized coordinates. Since these are time-dependent initial value problems, the Runge–Kutta method is used to solve the equations, which can provide an efficient and accurate time integration. This method is then used to predict the pipe vibration under the effects of tension, internal flow velocity, and wake oscillator excitation. During calculations, a fixed time step of Δ*τ* = 0.001 is adopted. Unless otherwise stated, the initial parameters are set according to Dai et al. [55], with ${\bar{\bar{v}}}_{i}={\dot{\bar{\bar{v}}}}_{i}={\bar{q}}_{i}={\dot{\bar{q}}}_{i}$ = 0.000025.

In the following discussion, the maximum response amplitude of the structure is denoted as *A_viv_* = $\underset{\tau }{\max}\left|\bar{v}\left(\xi ,\tau \right)\right|$, and the peak-to-peak oscillation amplitude is denoted as *A_mean_* = $\frac{1}{2}\left[\underset{\tau }{\max}\bar{v}\left(\xi ,\tau \right)-\underset{\tau }{\min}\bar{v}\left(\xi ,\tau \right)\right]$. *A_env_* = $\left\{\underset{\tau }{\max}\bar{v}\left(\xi ,\tau \right),\underset{\tau }{\min}\bar{v}\left(\xi ,\tau \right)\right\}$ represents the upper and lower bounds of the structural response, including the maximum and minimum amplitudes.

## 3. Model Validation and Convergence Analysis

### 3.1. Comparison Between the Full Coupled Model and the Reduced Model

To improve computational efficiency, the original formulation was simplified in Section 2.2. This subsection further verifies the validity of the adopted simplification.

**Figure 3:**
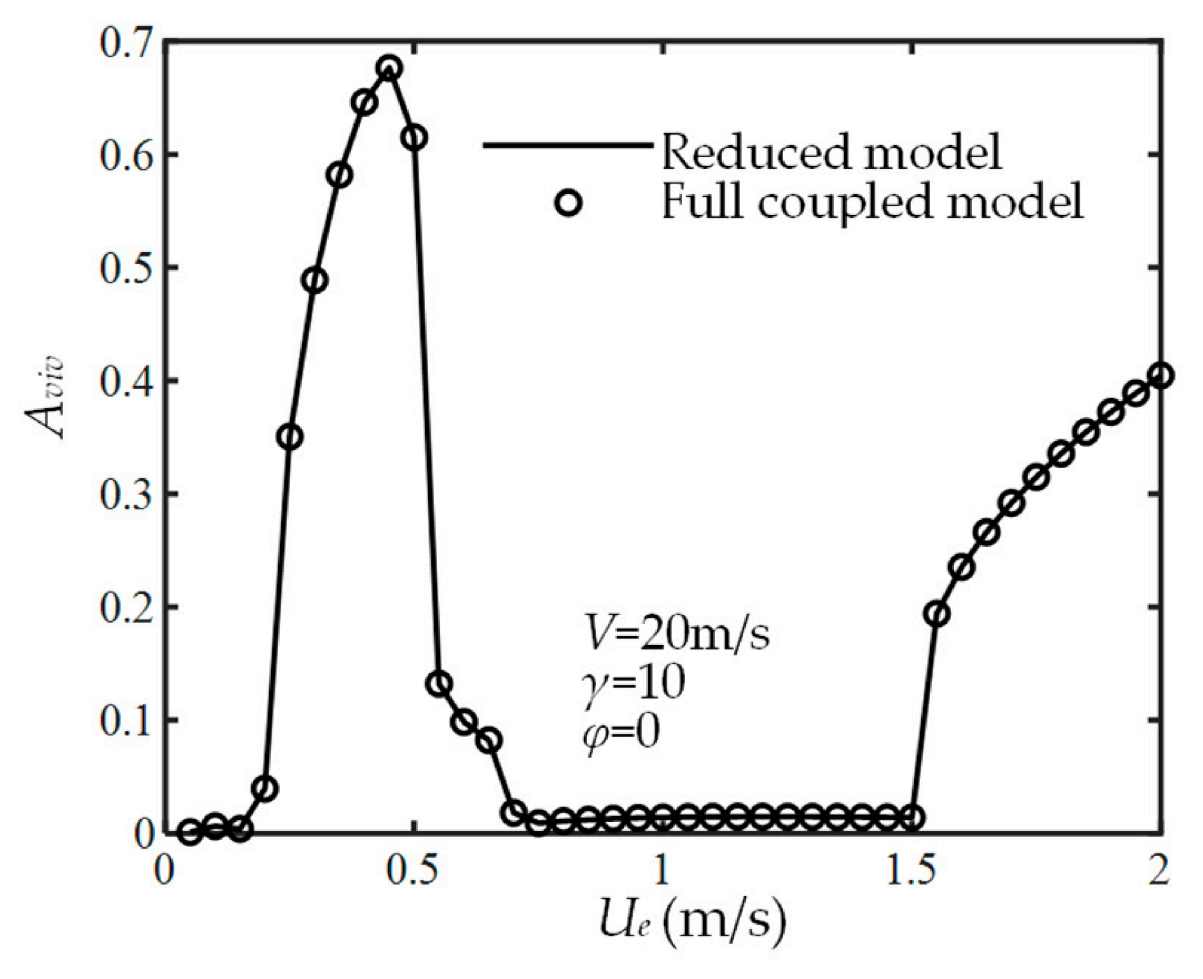
Comparison of response envelopes of a slightly curved pipe predicted by the fully coupled and reduced models.

Figure 3 presents a comparison between the results obtained from the fully coupled model and the reduced model. The fully coupled model represents the VIV responses calculated directly based on Equations (11) and (12), while the reduced model corresponds to the formulation derived from Equation (17). In this study, gravity is considered together with the corresponding initial static deformation. Therefore, to focus only on the effect of the model simplification, the gravity term in Equations (11), (12) and (17) is neglected in this subsection. An error parameter *e_or_* is defined to quantify the difference between the two models, which is expressed as

(25) $$e_{or}=\frac{{\left\|{\bar{v}}_{full}-{\bar{v}}_{red}\right\|}_{L^{2}}}{{\left\|{\bar{v}}_{full}\right\|}_{L^{2}}}.$$

The value of *e_or_* in Figure 3 is 0.0476%. The results in Figure 3 show good agreement between the two models, indicating that the reduced model can effectively capture the axial stretching effect caused by the bending vibration of the pipe.

### 3.2. Validations of the Present Model

**Figure 4:**
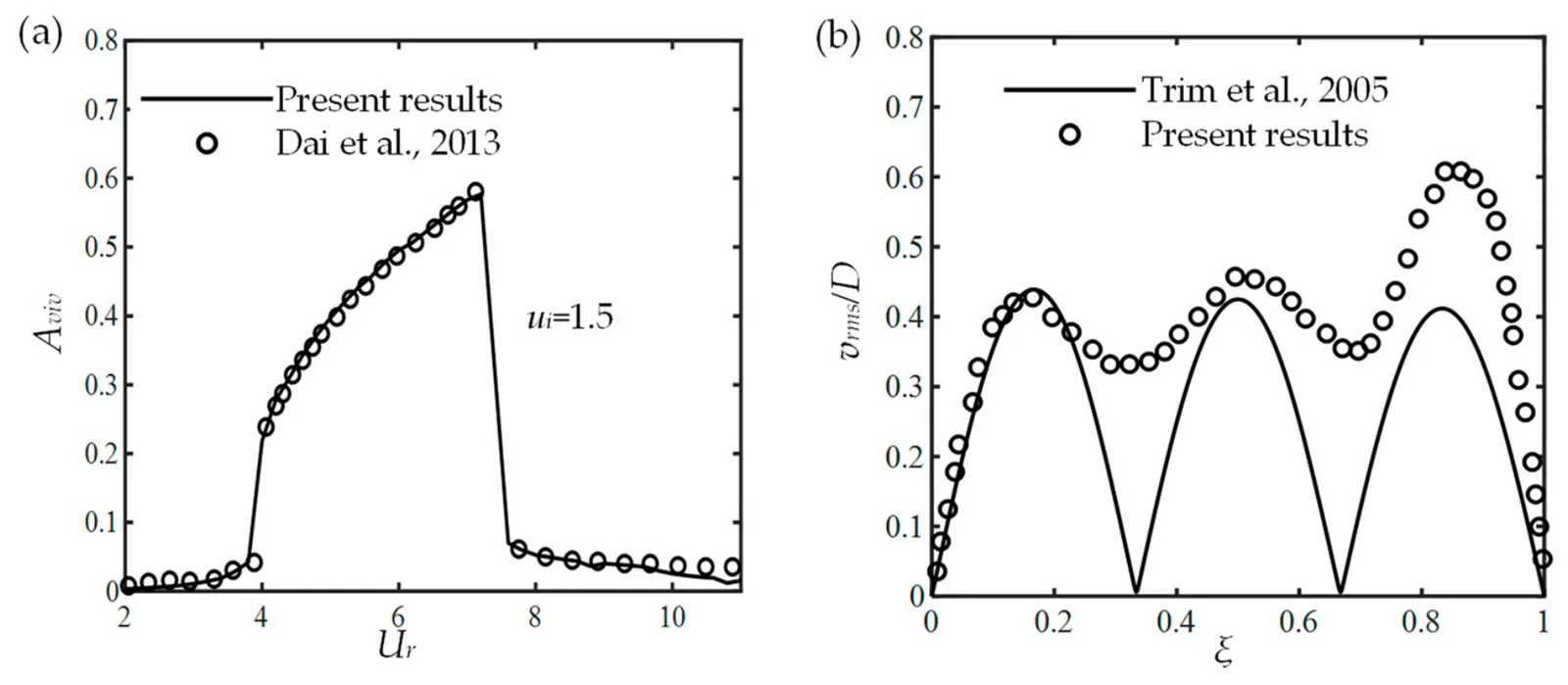
Comparisons of the present results with those reported by Dai et al. [55] in terms of (**a**) maximum response amplitude and by Trim et al. [63] in terms of (**b**) root mean square response amplitude.

Dai et al. [55] have investigated the VIV responses of an ideal horizontal straight fluid-conveying pipe under both subcritical and supercritical internal flow velocities. The structural and material parameters of the fluid-conveying pipe used in Dai et al. [55] are listed in Table 2, and the relationship between the maximum VIV response of the pipe and the external flow velocity is illustrated in Figure 4a. The horizontal axis represents the dimensionless external flow velocity, i.e., the reduced external flow velocity *U_r_* = 2π*U_e_*/(*ω*_1_*D*), where *ω*_1_ denotes the first-order angular frequency of the structure. It is noted that the present model accounts for the effect of initial imperfections, i.e., *φ*, as well as gravity and axial tension, corresponding to the sixth and seventh terms in Equation (17), which are not considered in the model reported by Dai et al. [55].

It can be seen from Figure 4a that, as the external flow velocity increases, the maximum transverse VIV response of the pipe first increases and then decreases. Within the range of 4 < *U_r_* < 7.8, lock-in resonance occurs. The results obtained by the proposed model also predict this phenomenon and show good agreement with those reported values [55].

Trim et al. [63] investigated the VIV responses of a straight pipe experimentally, and the relevant pipe parameters are summarized in Table 3. Figure 4b compares the root mean square (RMS) displacement responses in the cross-flow direction obtained from the experiment and the present model. The comparison indicates that the pipe response is dominated by the third mode, which agrees well with the prediction of the proposed model. Moreover, the calculated RMS responses show good agreement with the experimental results, validating the capability of the proposed model in predicting VIV responses.

**Figure 5:**
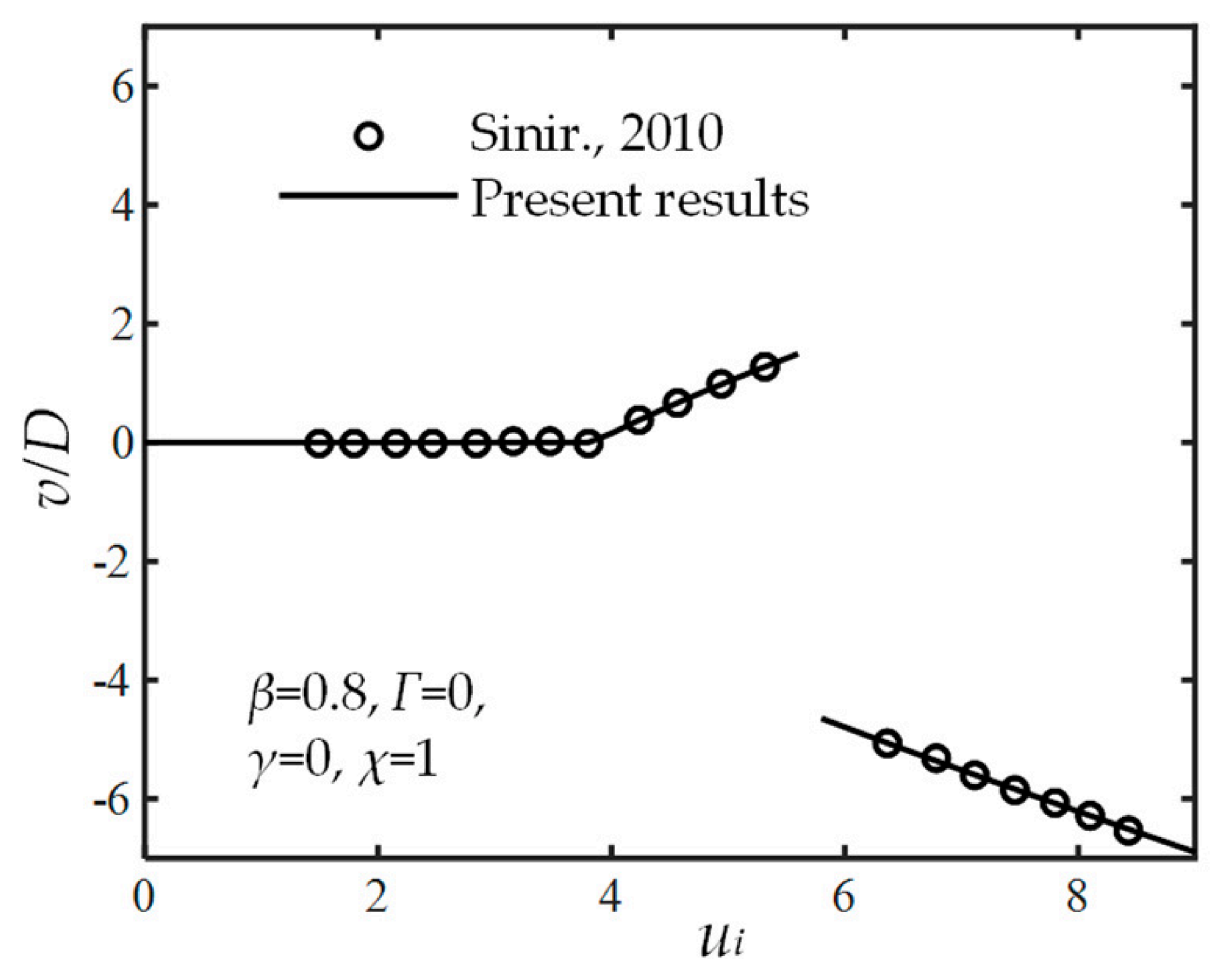
Comparison of the bifurcation diagrams of the slightly curved pipe between the present results and those reported by Sinir [64] at *ξ* = 0.5 with *φ* = sin (πx).

Regarding the structural responses of pipes with slight initial curvature, Sinir [64] established a dynamic model for a fluid-conveying pipe with initial deformation and investigated its bifurcation and chaotic behaviors. In that study, the pipe response was induced only by the internal flow, and the main focus was on the buckling behavior. In addition, the gravity effect, represented by the sixth term in Equation (17), was neglected. For a sinusoidal initial deformation, i.e., *φ* = sin (π*x*), the variation in the maximum buckling amplitude with *u_i_* is shown in Figure 5. Using the proposed model without the wake oscillator, the buckling behavior of the same pipe was calculated, and the results are also included in Figure 5 for comparison. It can be seen that when the dimensionless internal flow velocity *u_i_* exceeds 4, the pipe exhibits bifurcation in its transverse direction. It can also be seen from Figure 5 that the results obtained by the proposed model are in good agreement with those reported by Sinir [64].

**Figure 6:**
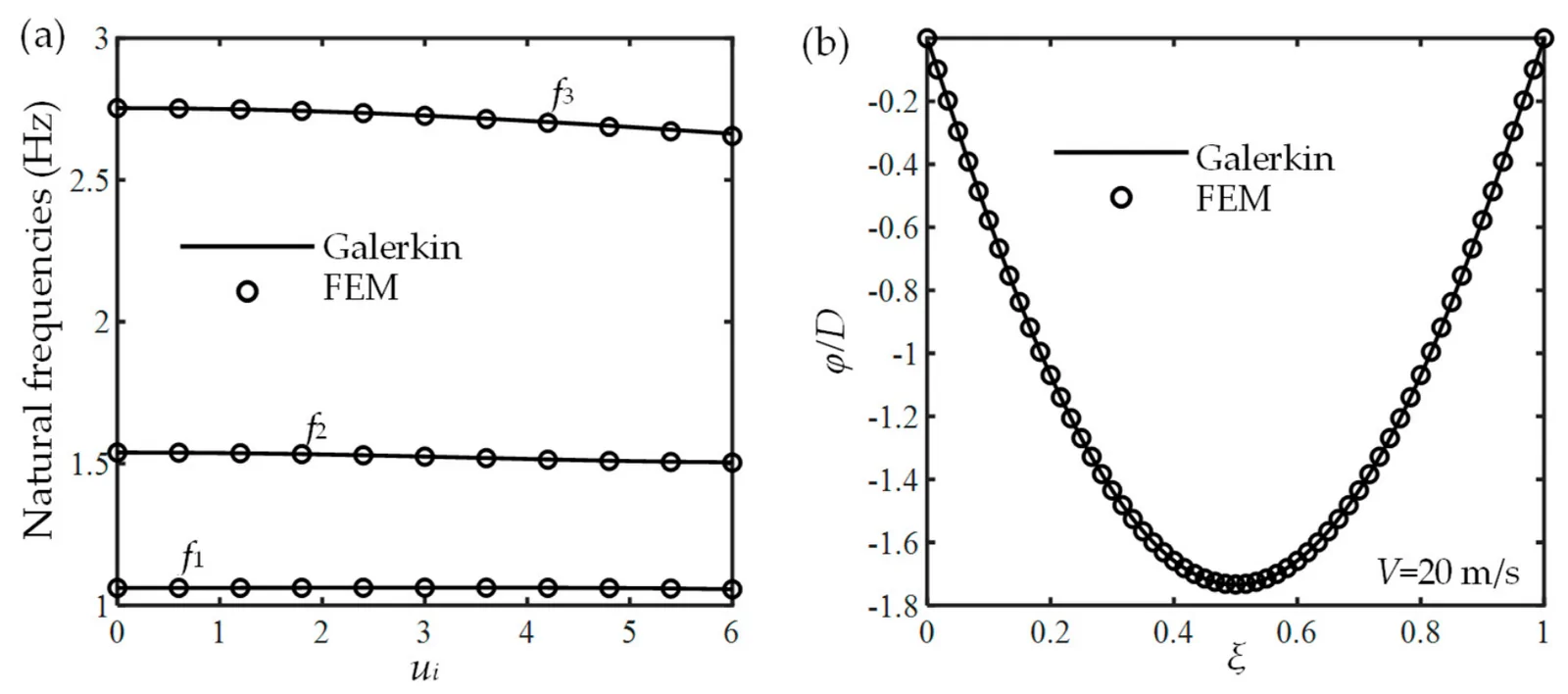
Comparison of the results obtained by the finite element method (FEM) and Galerkin method for *γ* = 10. (**a**) Natural frequencies and (**b**) initial static deformation.

To verify the accuracy of the calculated gravity-induced static configuration, the results obtained in this study are compared with those obtained by the finite element method, as shown in Figure 6a, which compares the natural frequencies calculated using Equation (29), while Figure 6b compares the initial static deformations obtained by the Galerkin and finite element methods. The calculation procedure based on the Galerkin method is described in Section 4. The finite element discretization of Equation (26) follows the procedure given in Ref. [65]. Since gravity is a constant load, the time-dependent terms are neglected in the finite element analysis, and Equation (26) is solved as a static problem. Therefore, Equation (26) is reduced to ${\left[K\right]}_{e}v_{e}={\left[F\right]}_{e}$, where ${\left[K\right]}_{e}$ is the global stiffness matrix, ${\left[F\right]}_{e}$ is the load vector. Solving this equation gives the gravity-induced deformation distribution along the pipe. It can be observed from Figure 6 that the results obtained by the present method agree well with the finite element results. The maximum sag in Figure 6b is *φ*/*D* = 1.7341 from the Galerkin method and *φ*/*D* = 1.7335 from the finite element method. The relative difference is negligible, demonstrating the reliability of the calculated gravity-induced equilibrium configuration.

From the above comparisons, it can be concluded that the model established and the computational program developed in this study are suitable and reliable for predicting the VIV behaviors of the slightly curved pipe containing the internal flow and subject to varying tension.

### 3.3. Truncate Order

This study employs the Galerkin method to solve the established dynamic equations. Before solving the equations, it is necessary to determine a reasonable truncation order *N*. A larger *N* yields higher computational accuracy but lower efficiency, whereas a smaller *N* results in higher computational efficiency but lower accuracy.

**Figure 7:**
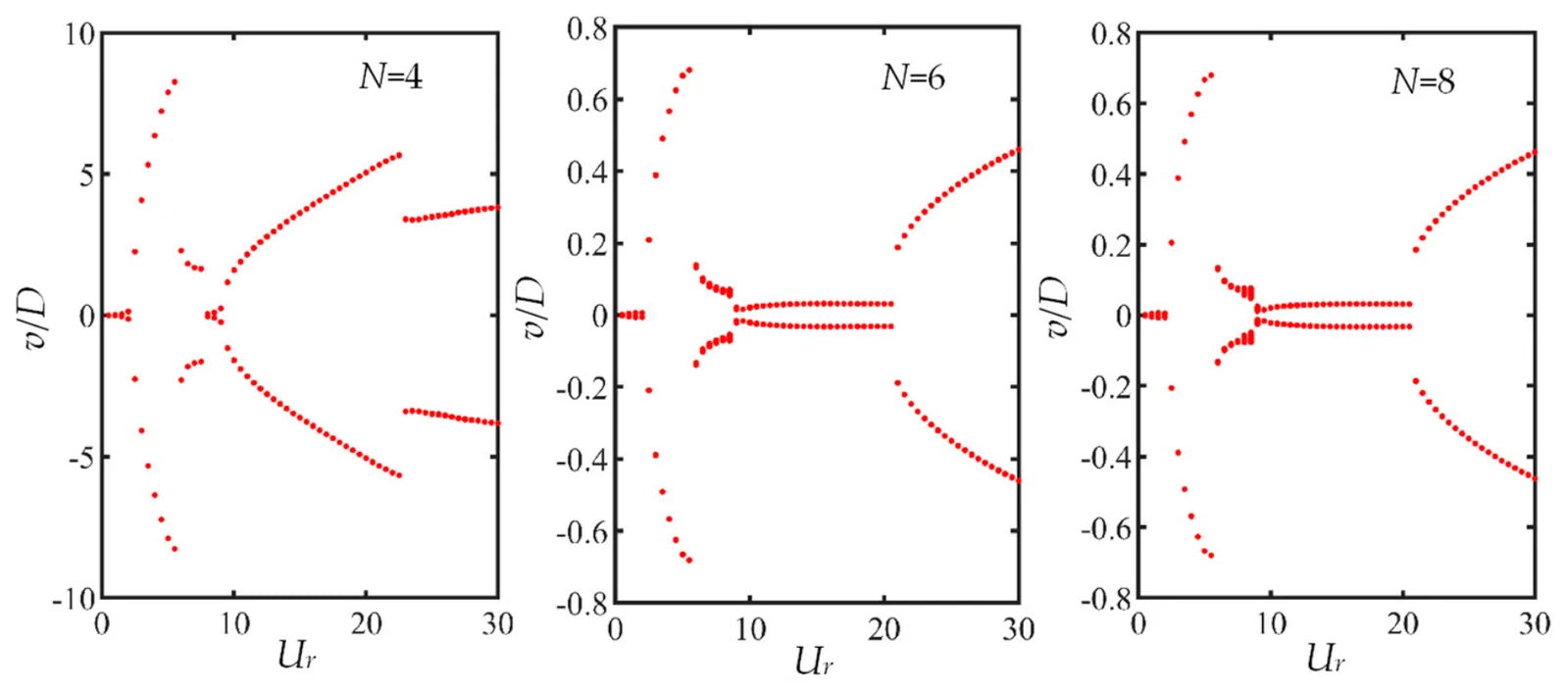
Bifurcation diagrams of the pipe for different truncation mode orders at *V* = 40 m/s, *γ* = 10, *φ* = 0 and *ξ* = 0.5.

**Figure 8:**
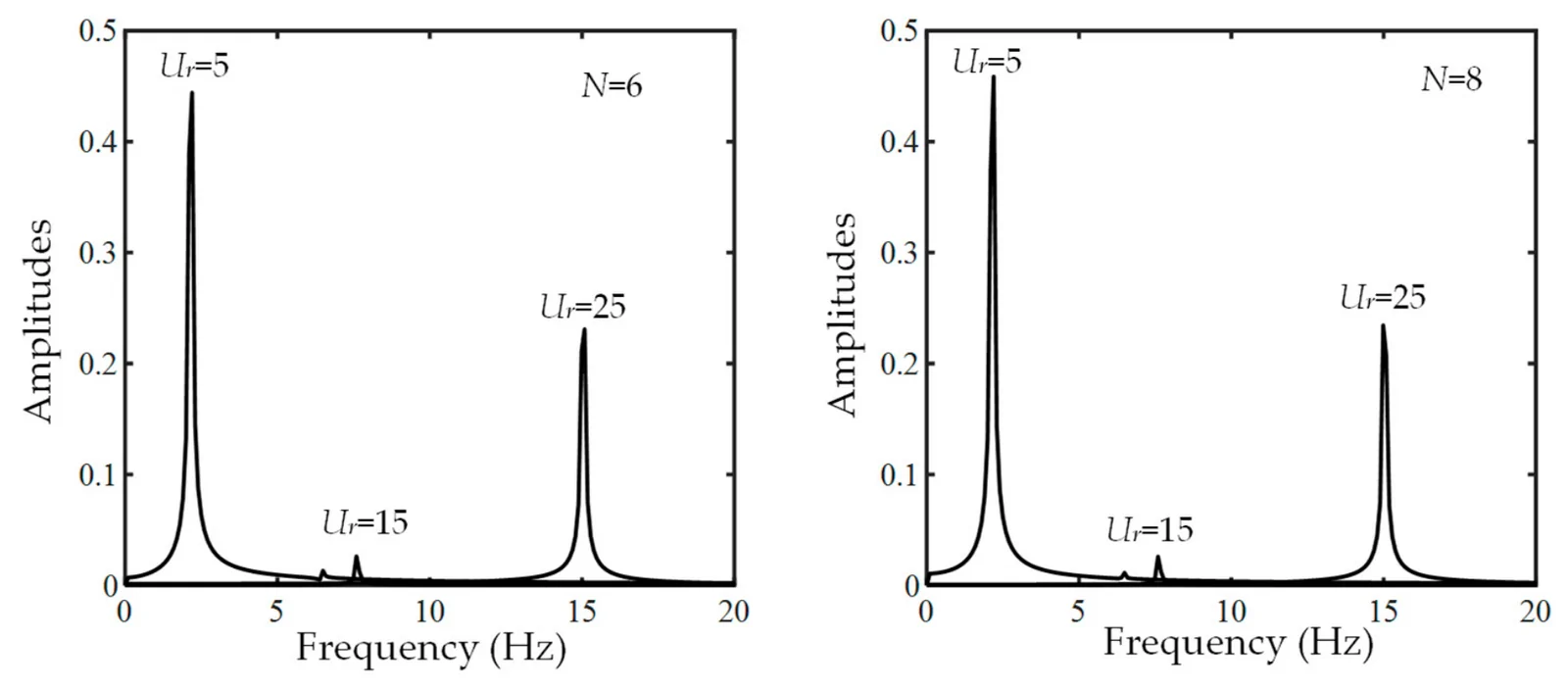
Frequency spectra of the pipe for different truncation mode orders at *V* = 40 m/s, *γ* = 10, *φ* = 0 and *ξ* = 0.5.

Figure 7 presents the bifurcation diagrams of the midpoint response of the straight pipe for different truncation mode orders. The results are calculated based on Equation (17), with the gravity term neglected. The internal and external flow velocities are set as *V* = 40 m/s and *U_e_* = 2 m/s, respectively. The nondimensional tension is set to *γ* = 10. This condition corresponds to the maximum response mode discussed in the following sections. It can be observed from Figure 7 that the bifurcation behaviors obtained with *N* = 6 and *N* = 8 are identical. However, significant differences are observed between *N* = 4 and *N* = 6 or 8 in the range of 22 < *U_r_* < 30. The response spectra at the midpoint of the pipe for *N* = 6 and 8 are further presented in Figure 8. It can be observed that both cases exhibit the same three peak frequencies, indicating that the two truncation orders provide consistent predictions of the dynamic characteristics. Therefore, *N* = 6 is adopted for the subsequent analysis.

### 3.4. Temporal Convergence

Adaptive time-stepping strategies based on instantaneous oscillatory or characteristic time scales can improve computational efficiency while maintaining numerical accuracy. Such strategies have been successfully applied in other multiscale wave-evolution problems [66]. In this study, the Runge–Kutta method with a fixed time step is employed to solve the dynamic equations. This choice is made because a relatively small fixed time step is sufficient to obtain accurate results with acceptable computational efficiency for the present VIV problem.

A smaller time step can improve computational accuracy but increases the computational cost and data storage requirements. In contrast, a larger time step can reduce computational time but may lead to inaccurate results. Therefore, an appropriate time step is required to balance accuracy and efficiency. In this subsection, the time-step convergence is investigated.

**Figure 9:**
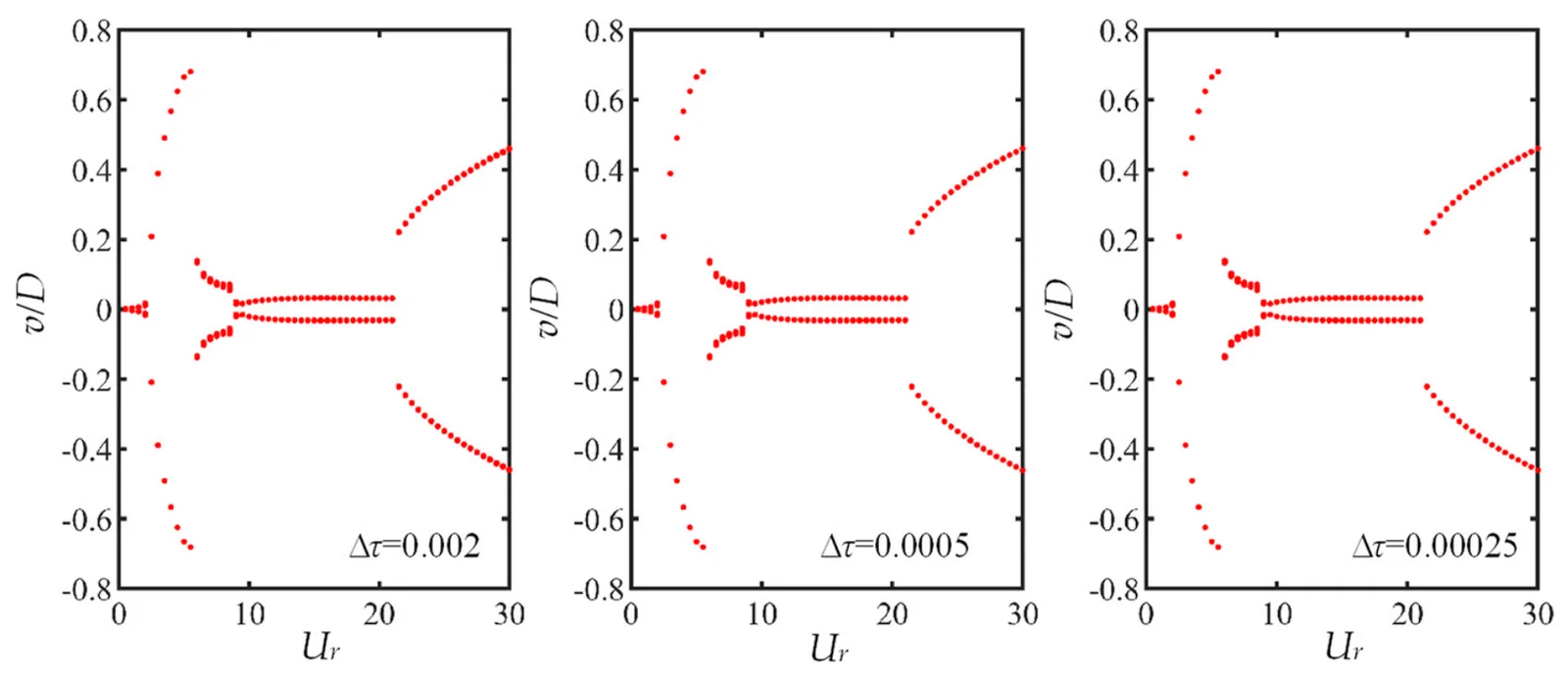
Bifurcation diagrams of the pipe response for different time steps at *V* = 40 m/s, *γ* = 10, *φ* = 0 and *ξ* = 0.5.

**Figure 10:**
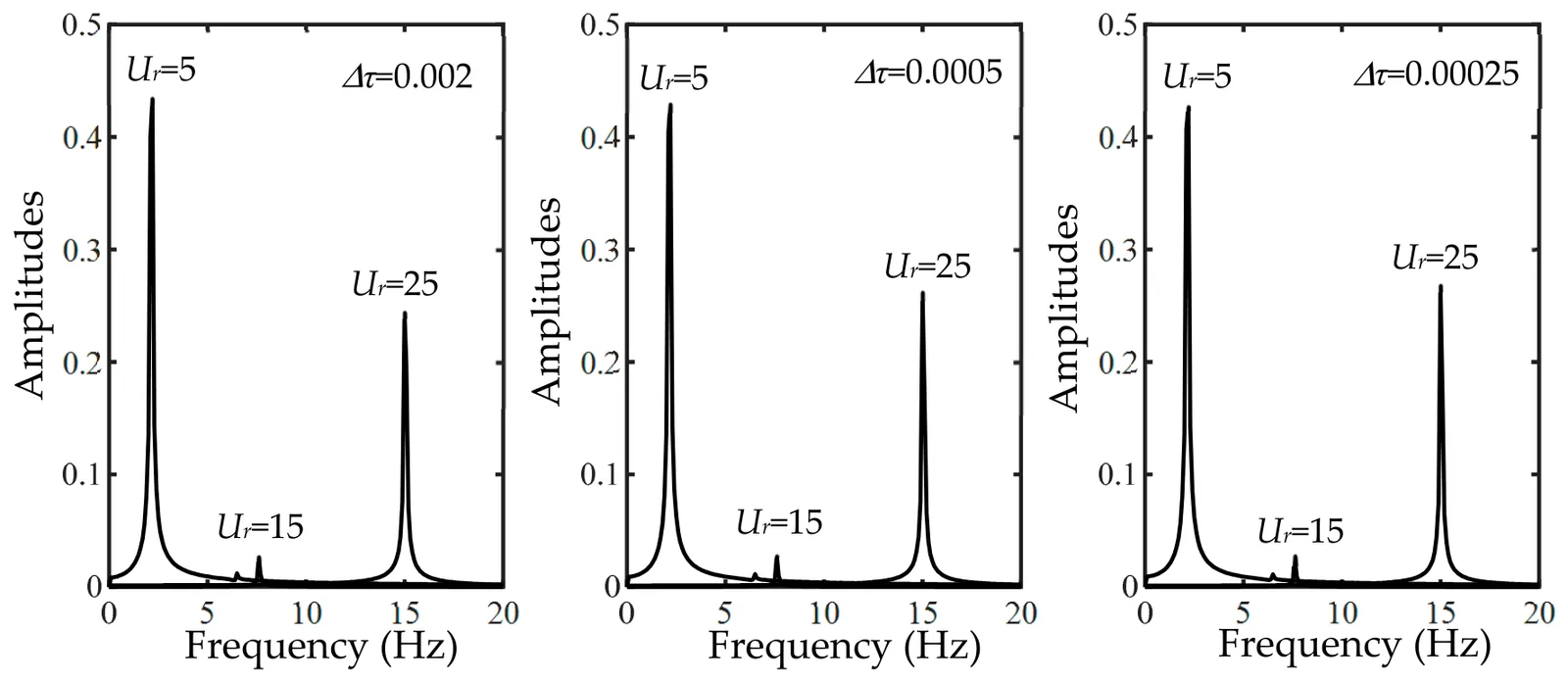
Frequency spectra of the pipe response for different time steps at *V* = 40 m/s, *γ* = 10, *φ* = 0 and *ξ* = 0.5.

**Figure 11:**
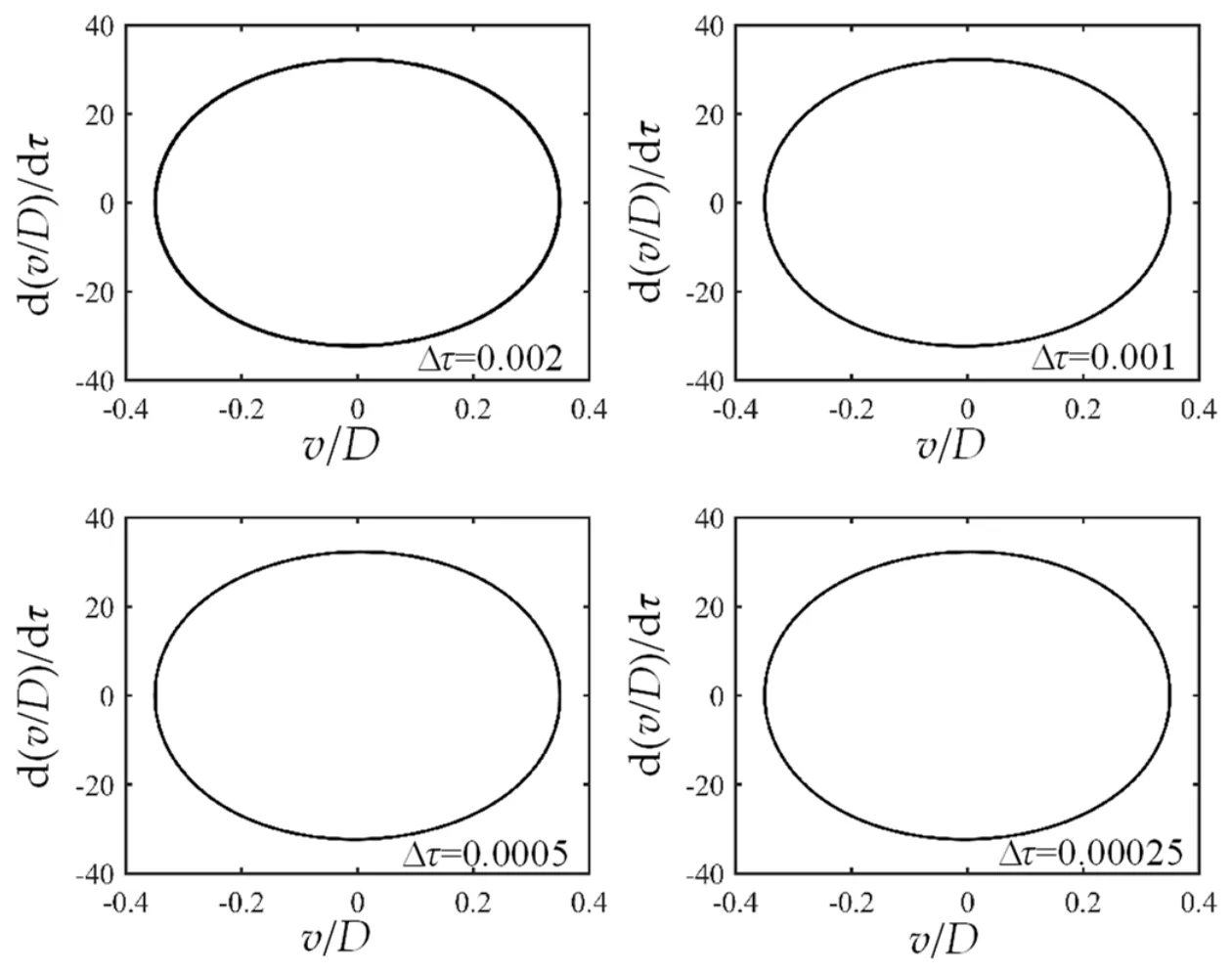
Phase portraits of the pipe response for different time steps at *V* = 40 m/s, *γ* = 10, *ξ* = 0.5, *φ* = 0 and *U_r_* = 25.

The results in Figure 7 and Figure 8 are further discussed below. A time step of Δ*τ* = 0.001 is used in Figure 7 and Figure 8. The bifurcation diagrams and frequency spectra for time steps of 0.002, 0.0005 and 0.00025 are further presented in Figure 9 and Figure 10. The truncation mode order is set to *N* = 6 in all cases. As shown in Figure 7, Figure 8, Figure 9 and Figure 10, the frequency spectra are almost identical for time steps of 0.002, 0.001, 0.0005, and 0.00025. The phase portraits of the midpoint response at *U_r_* = 25 for the four time steps are shown in Figure 11. According to Figure 11, the pipe shows similar motion patterns at the four time steps. To quantify the time convergence, an error parameter $e_{\Delta \tau }={\left\|{\left({\bar{v}}_{\Delta \tau }\right)}_{rms}-{\left({\bar{v}}_{ref}\right)}_{rms}\right\|}_{L^{2}\left(0,T\right)}$ is defined, where ${\bar{v}}_{\Delta \tau }$ represents the root mean square displacement calculated with a time step of Δ*τ*, and ${\bar{v}}_{ref}$ represents the root mean square displacement obtained from the reference solution using Δ*τ* = 0.0001. The comparison results are listed in Table 4. In Table 4, $p=\log_{2}\left(e_{\Delta \tau }/e_{0.5\Delta \tau }\right)$. The results in Table 4 indicate that a time step of 0.001 is sufficient for the present calculations.

## 4. Results and Discussions

For pipes with gravity-induced sagging, the initial deflection may vary with axial tension and internal flow velocity. Therefore, assuming a fixed sinusoidal initial configuration may lead to inaccurate results in practical applications. Unlike previous VIV models, the following calculations consider the effects of different axial tensions and internal flow velocities on the gravity-induced equilibrium configuration. The structural and material parameters of the pipe used to make the predictions are shown in Table 5.

First, the effects of tension and internal flow velocity on the gravity-induced initial curvature are investigated. The obtained deflection shapes are then used in the subsequent VIV analyses. Referring to the ranges of internal flow velocity reported in previous studies [68,69], the present study considers dimensional internal flow velocities of *V* = 0 m/s, 20 m/s and 40 m/s. The initial static deformation of the pipe is obtained by the following steps:

(1) First, the initial equilibrium configuration of the pipe under gravity, denoted as $\bar{\varphi }$, is determined using the straight pipe model. By neglecting the hydrodynamic terms in Equation (18) and excluding the wake oscillator equation, i.e., Equation (19), the governing equation of the straight pipe under gravity is obtained as

(26) $$\ddot{\bar{\varphi }}+2u_{i}\sqrt{\beta }{\dot{\bar{\varphi }}}^{\prime }+u_{i}^{2}{\bar{\varphi }}^{\prime \prime }+\sigma_{s}\dot{\bar{\varphi }}+{\bar{\varphi }}^{⁗}+\Gamma -\gamma {\bar{\varphi }}^{\prime \prime }-\chi {\bar{\varphi }}^{\prime \prime }\int_{0}^{1}\left(\frac{1}{2}{{\bar{\varphi }}^{\prime }}^{2}\right)d\xi =0,$$

It is noted that, after neglecting the gravity term *Γ*, Equation (26) becomes identical to the structural equation presented in Ref. [55]. The formulation of the gravity term in Equation (26) is adopted from Ref. [52].

(2) Equation (26) is discretized using the Galerkin method, and the resulting discretized equations are iteratively solved using the Runge–Kutta method. The simply supported boundary conditions are maintained throughout the calculation. Therefore, the same trial functions can still be used in the Galerkin discretization.

(3) Since the gravity force is constant, the solution of Equation (26) gradually converges to a constant value. This converged solution represents the gravity-induced initial static deformation of the pipe for each combination of axial tension and internal flow velocity. Equation (26) is solved independently until the deformation remains unchanged with time. At this stage, the time-dependent terms in Equation (26) become zero, i.e., $\ddot{\bar{\varphi }}=\dot{\bar{\varphi }}=0$. The obtained Galerkin representation of the equilibrium configuration is directly used in the subsequent calculations.

It should be noted that, when Equations (21) and (22) are used to predict the dynamic behavior of the slightly curved pipe, the initial static deformation must be determined from Equation (26) for each combination of axial tension and internal flow velocity. By substituting Equation (26) into Equations (21) and (22), the actual governing equations used for the dynamic analysis, considering the gravity-induced initial deformation, are obtained as

$${\ddot{\bar{v}}}_{d}+2u_{i}\sqrt{\beta }{{\dot{\bar{v}}}_{d}}^{\prime }+u_{i}^{2}{{\bar{v}}_{d}}^{\prime \prime }+\left(\sigma_{s}+\sigma_{f}\right){\dot{\bar{v}}}_{d}+{{\bar{v}}_{d}}^{⁗}$$

(27) $$-\gamma {{\bar{v}}_{d}}^{\prime \prime }-\chi {\bar{\varphi }}^{\prime \prime }\int_{0}^{1}\left(\frac{1}{2}{{{\bar{v}}_{d}}^{\prime }}^{2}+{\bar{\varphi }}^{\prime }{{\bar{v}}_{d}}^{\prime }\right)d\xi -\chi {{\bar{v}}_{d}}^{\prime \prime }\int_{0}^{1}\left(\frac{1}{2}{{\bar{\varphi }}^{\prime }}^{2}+\frac{1}{2}{{{\bar{v}}_{d}}^{\prime }}^{2}+{\bar{\varphi }}^{\prime }{{\bar{v}}_{d}}^{\prime }\right)d\xi =\alpha q.$$

(28) $$\ddot{q}+\varepsilon_{y}\Omega_{s}\left(q^{2}-1\right)\dot{q}+\Omega_{s}^{2}q=A_{y}{\ddot{\bar{v}}}_{d},$$

**Figure 12:**
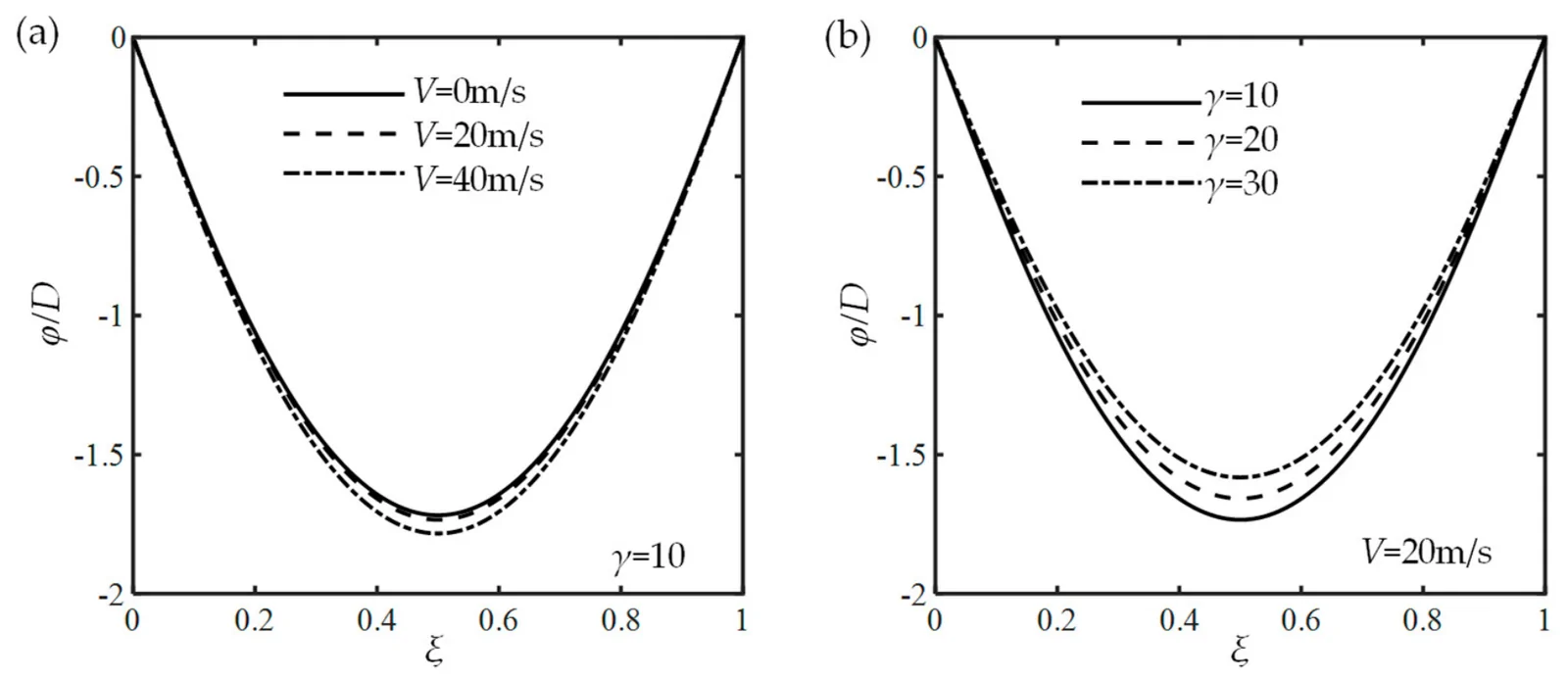
Initial static deformation of the fluid-conveying pipe under varying (**a**) internal velocities and (**b**) tensions.

Figure 12 presents the nondimensional deflections along the pipe under different internal flow velocities and tensions. As shown in Figure 12a, increasing the internal flow velocity increases the initial static deflection of the pipe. This is because a higher internal flow velocity reduces the effective stiffness of the structure, resulting in larger deformation. In contrast, increasing the tension decreases the initial static deflection, as shown in Figure 12b. This is attributed to the fact that higher tension increases the structural stiffness and therefore reduces the deformation.

**Figure 13:**
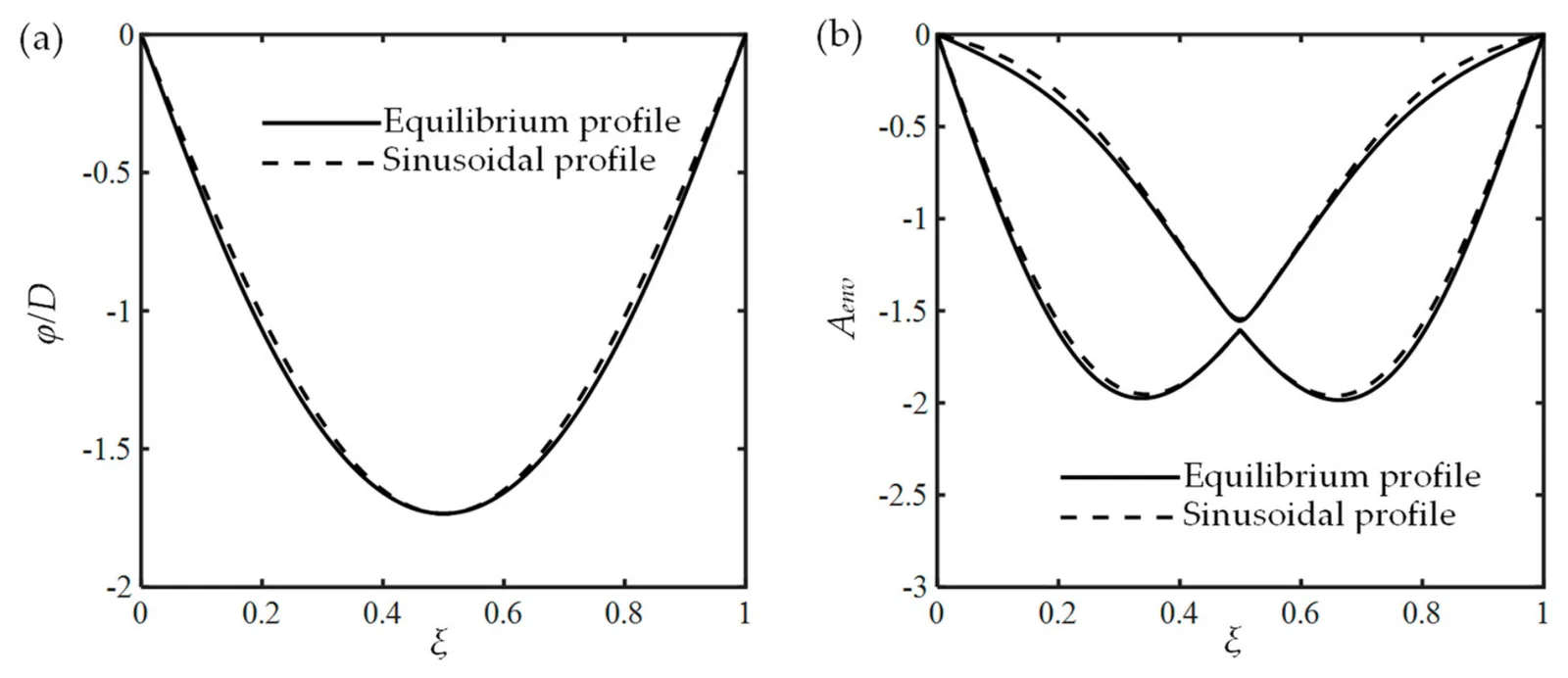
Comparison between (**a**) the calculated equilibrium profile and the prescribed sinusoidal profile with the same maximum sag, and (**b**) the corresponding responses of the pipe with the two profiles at *V* = 20 m/s and *γ* = 10.

It can be observed from Figure 12 that the gravity-induced initial static deformation is approximately sinusoidal, with the maximum deflection occurring near the midpoint of the pipe. However, the deformation is not exactly sinusoidal. Taking the case of *V* = 20 m/s and *γ* = 10 as an example, the equilibrium configuration obtained from the Galerkin representation is compared with a prescribed sinusoidal profile with the same maximum sag. The corresponding VIV responses of the pipe with the two configurations are also compared, as shown in Figure 13. The modal coefficients and relative contributions in the Galerkin representation of the gravity-induced static deformation are listed in Table 6.

It can be observed from Figure 13 that the gravity-induced equilibrium configuration obtained from the calculation has larger deflections than the prescribed sinusoidal profile near the locations of *ξ* = 0.25 and 0.75. The corresponding VIV responses at these locations are also larger than those predicted using the sinusoidal profile.

### 4.1. Natural Frequencies of the Pipe

For convenience, the statement “considering gravity effect” refers to the results obtained by solving the governing equations derived from Equations (18) and (19) while neglecting the gravity terms. The changes in the natural frequencies of the pipe before and after considering the gravity effect are first discussed. The calculation results are then used in the following analyses. By neglecting the external force and nonlinear terms in Equation (27), the equation for calculating the natural frequencies of the pipe is expressed as

(29) $${\ddot{\bar{v}}}_{d}+2u_{i}\sqrt{\beta }{{\dot{\bar{v}}}_{d}}^{\prime }+u_{i}^{2}{{\bar{v}}_{d}}^{\prime \prime }+\sigma_{s}{\dot{\bar{v}}}_{d}+{{\bar{v}}_{d}}^{⁗}-\gamma {{\bar{v}}_{d}}^{\prime \prime }-\chi {\bar{\varphi }}^{\prime \prime }\int_{0}^{1}{\bar{\varphi }}^{\prime }{{\bar{v}}_{d}}^{\prime }d\xi -\frac{\chi {{\bar{v}}_{d}}^{\prime \prime }}{2}\int_{0}^{1}{{\bar{\varphi }}^{\prime }}^{2}d\xi =0.$$

The seventh and eighth terms on the left-hand side of Equation (29) represent the effect of gravity-induced initial deformation.

**Figure 14:**
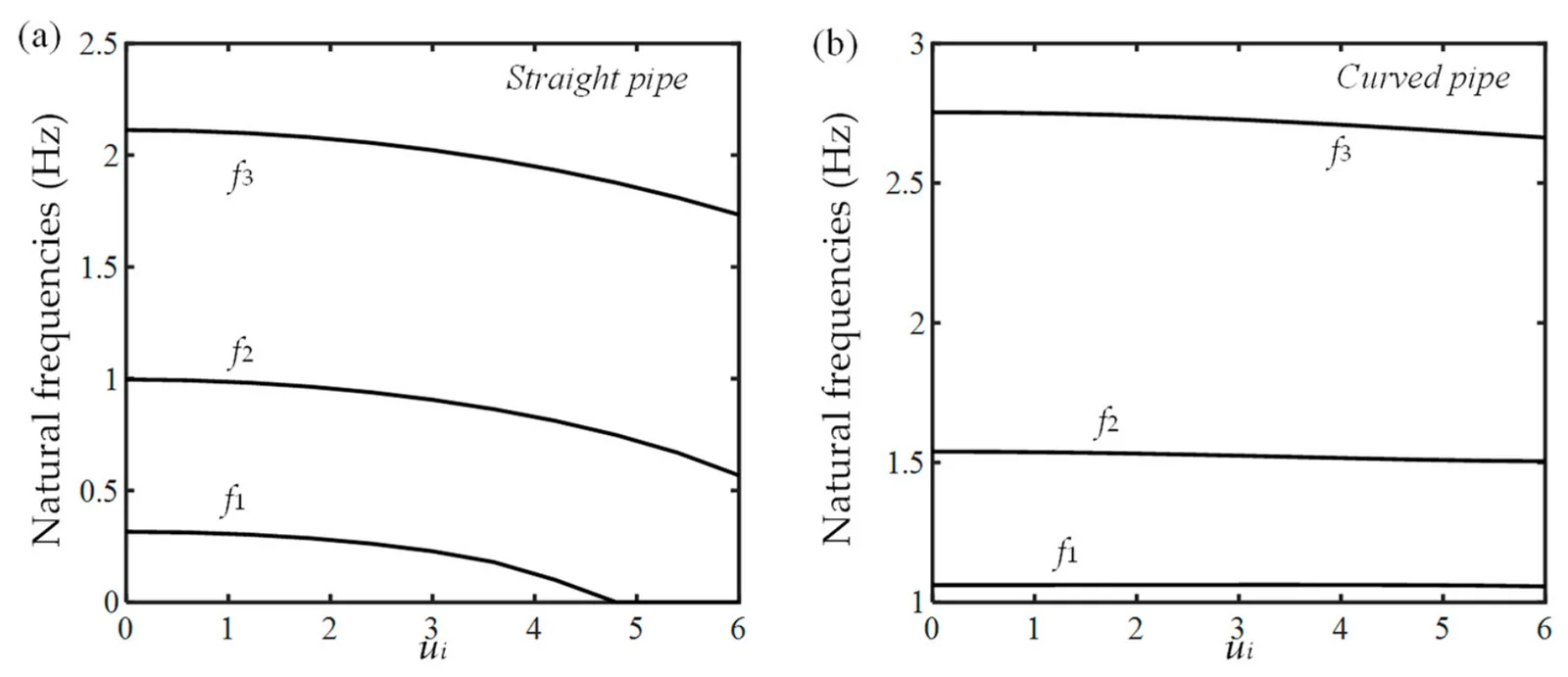
Variations in the first three natural frequencies of (**a**) the straight pipe and (**b**) the slightly curved pipe with internal flow velocity at *γ* = 10.

Figure 14 shows the variation in the pipe’s natural frequencies with the nondimensional internal flow velocity and tensions, before and after considering the gravity effect. It can be seen from Figure 14 that when *φ* = 0, i.e., for a straight pipe, the natural frequencies of the structure tend to decrease with increasing internal flow velocity. When the nondimensional internal flow velocity *u_i_* = 4.8, the first natural frequency of the structure approaches zero. At this point, the internal flow velocity is close to the critical internal flow velocity, and the structure is approaching an unstable state. This paper mainly focuses on the dynamic responses of the pipe under steady operating conditions. Therefore, the maximum internal flow velocity considered in the subsequent calculations is limited to *u_i_* = 4.8. Compared with the straight pipe, the natural frequencies increase when the gravity-induced initial curvature is considered. This increase is attributed to the additional stiffness introduced by the initial curvature. Specifically, the seventh and eighth terms in Equation (29) contribute to the geometric stiffness and consequently increase the natural frequencies of the pipe.

It can also be observed from Figure 14 that the gravity-induced initial configuration affects not only the first natural frequency but also the higher-order frequencies. The natural frequencies are obtained by discretizing Equation (29) using the Galerkin method and solving the corresponding eigenvalue problem. The trial functions used in the Galerkin discretization are given as $\phi_{i}=\sqrt{2}\sin(i\pi \xi )$. For a prescribed first-order sinusoidal imperfection, the modal orthogonality condition in Equation (24) indicates that the initial deformation *φ* only affects the first natural frequency. This has been verified in Xu et al. [53]. However, the gravity-induced configuration obtained in this study has a different spatial distribution and can therefore affect higher-order frequencies.

Figure 13 compares the equilibrium configuration obtained using the Galerkin representation with the prescribed sinusoidal profile. The corresponding first three natural frequencies of the two configurations are further compared in this subsection. For the Galerkin-based configuration, the first three natural frequencies are 1.0619 Hz, 1.5354 Hz, and 2.7471 Hz. For the prescribed sinusoidal profile, the corresponding values are 1.0456 Hz, 1.5176 Hz, and 2.7213 Hz. These results further demonstrate that the gravity-induced equilibrium configuration obtained in this study is different from the ideal sinusoidal profile.

### 4.2. Effect of External Flow Velocity

In the present study, the nondimensional external flow velocity, i.e., the reduced velocity *U_r_*, is used to describe the variation in the VIV responses with external flow velocity. Combining the expression for *U_r_* with Equation (10), the relationship between the vortex shedding frequency *ω_s_* and the structure’s first natural angular frequency *ω*_1_ can be obtained as follows

(30) $$\omega_{s}=U_{r}S_{t}\omega_{1},$$

**Figure 15:**
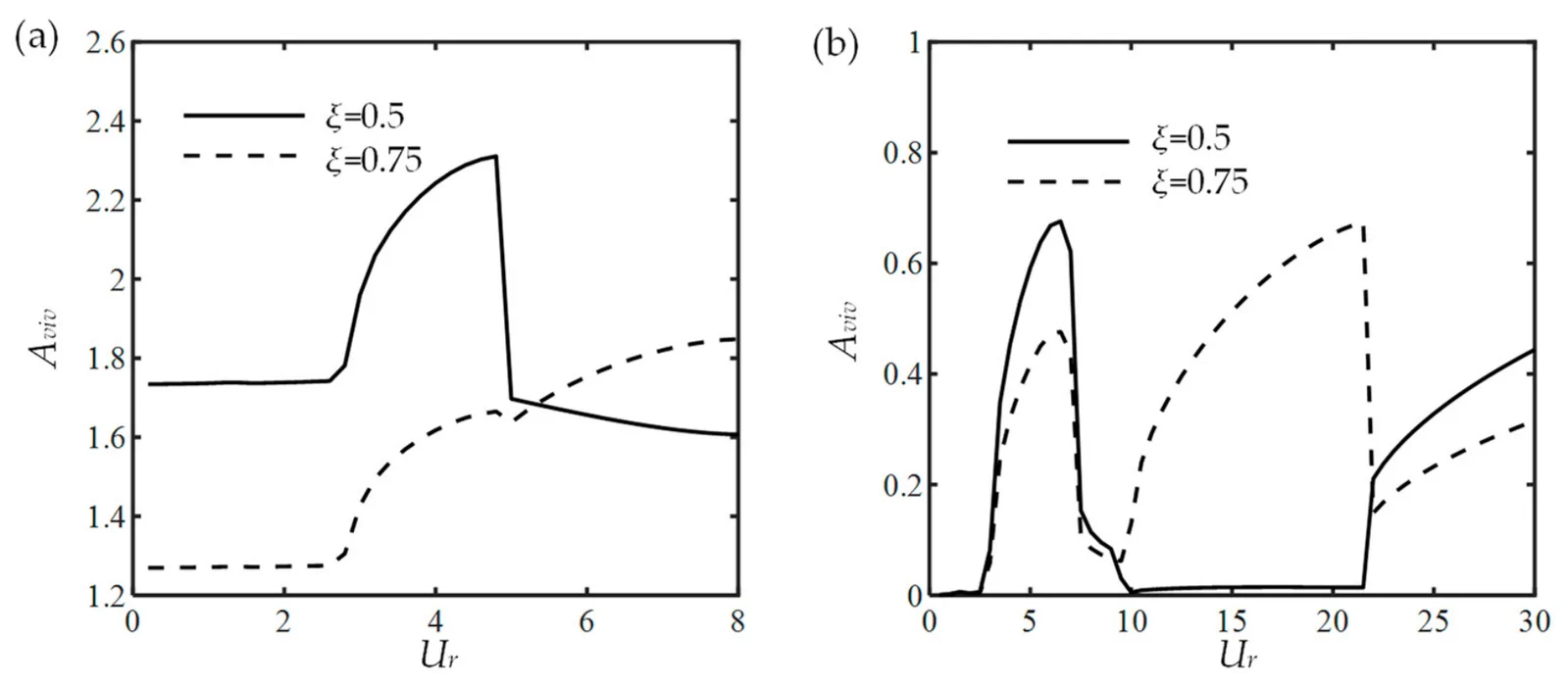
Variation in the maximum VIV responses of the slightly curved pipe with the reduced external flow velocity when *V* = 20 m/s and *γ* = 10. (**a**) Considering gravity effect, (**b**) without considering gravity effect.

From Equation (30), it can be seen that when *U_r_S_t_* = 1, the vortex shedding frequency equals the structure’s first natural angular frequency, and the structure will experience lock-in resonance. In the present study, the common value of *S_t_* = 0.2 is adopted in the calculations; therefore, when the reduced external flow velocity *U_r_* is near 5, the pipe will experience lock-in resonance. The theoretical analysis above agrees with the calculation results presented in Figure 15, which shows the maximum VIV responses under different reduced velocities. Two typical locations along the pipe (*ξ* = 0.5 and 0.75) are selected to evaluate the maximum VIV response. The results presented in Figure 15a account for the gravity effect, whereas those in Figure 15b are obtained for a straight pipe without gravity effect.

It should be noted that the maximum values of the horizontal axes in Figure 15 all correspond to a dimensional external flow velocity of approximately 2 m/s, although they show different reduced velocities. According to the definition of the reduced external flow velocity *U_r_*, the pipe’s natural frequencies differ before and after considering the gravity effect. As a result, the same dimensional external flow velocity *U_e_* leads to different values of *U_r_*, which in turn leads to different horizontal-axis ranges in Figure 15.

It is shown in Figure 15 that as *U_r_* increases from zero, the maximum VIV response of the pipe with gravity effect starts from a nonzero value, whereas that of the horizontal straight pipe starts from zero. This difference is caused by the gravity-induced initial curvature of the pipe. It can be seen from Figure 15 that when 3.8 < *U_r_* < 4.5, the case with gravity effect exhibits first-order lock-in resonance, while the second-order lock-in resonance occurs when 5 < *U_r_* < 8.

**Figure 16:**
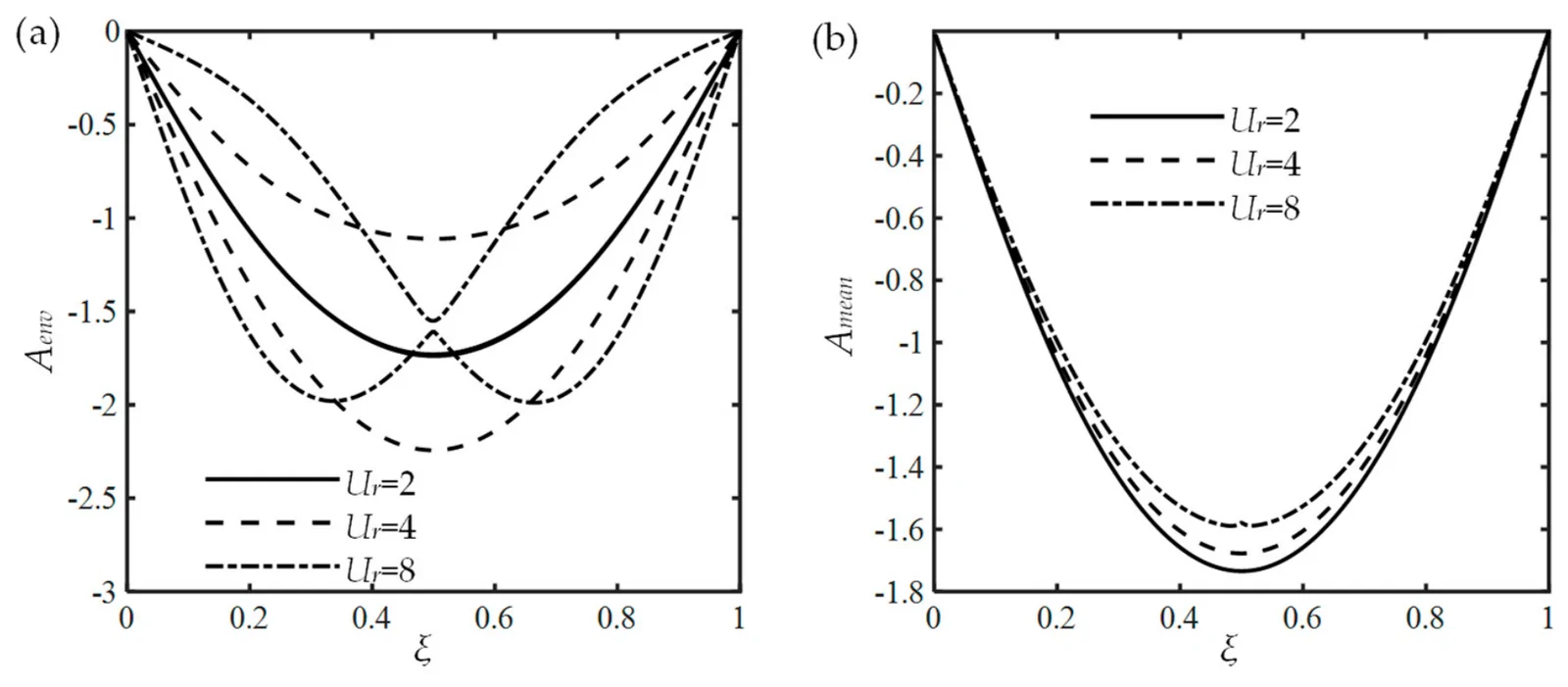
VIV responses of the slightly curved pipe under different reduced external flow velocities when *γ* = 10 and *V* = 20 m/s. (**a**) Response envelopes and (**b**) average response amplitudes.

The response envelope and the averaged responses of the pipe considering the gravity effect are shown in Figure 16. Figure 16a shows that during the second-order lock-in region, the midpoint of the pipe is close to the valley of the envelope, while the location at *ξ* = 0.75 is close to the crest. Therefore, for 6 < *U_r_* < 8, the maximum VIV response at *ξ* = 0.75 is larger than that at *ξ* = 0.5, as shown in Figure 15. From Figure 15b, it is shown that the straight pipe undergoes the first-order lock-in resonance when 4 < *U_r_* < 8 and the second-order lock-in resonance when 10 < *U_r_* < 22.

Based on the results in Figure 15a, three typical operating conditions (*U_r_* = 2, 4 and 8) are selected to further calculate the response envelopes and average oscillation amplitudes, as shown in Figure 16. As shown in Figure 15 and Figure 16, when *U_r_* increases from 2 to 4, the averaged deflection of the pipe considering the gravity effect decreases, while the oscillation amplitude and maximum response increase. The pipe eventually enters the first-order lock-in region. When *U_r_* further increases from 4 to 8, a mode transition occurs when the gravity effect is considered, as shown in Figure 16. This transition is accompanied by a significant decrease in the averaged deflection.

**Figure 17:**
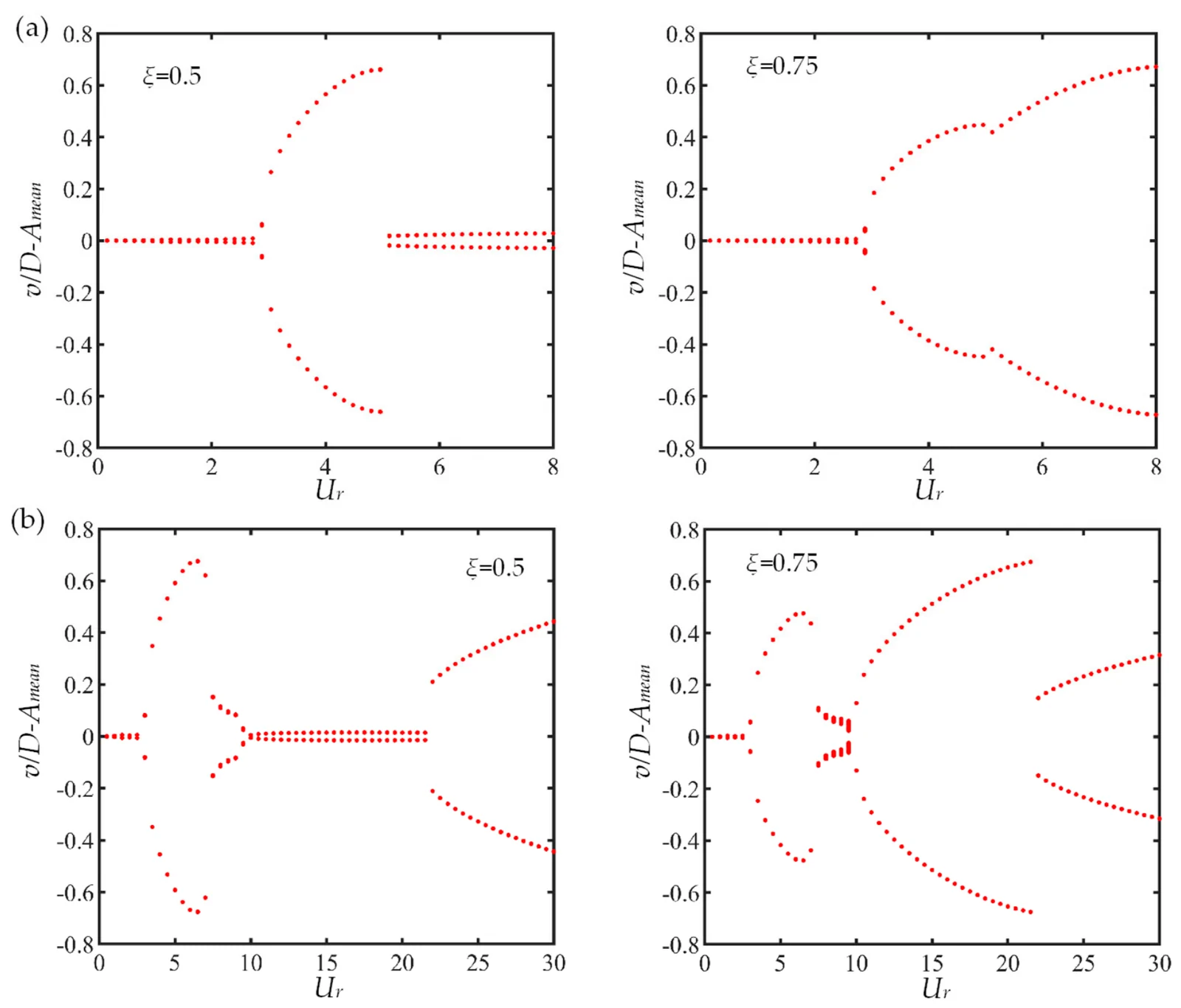
Bifurcation diagrams of the slightly curved pipe with the reduced external flow velocity when *γ* = 10 and *V* = 20 m/s. (**a**) Considering gravity effect and (**b**) without considering gravity effect.

**Figure 18:**
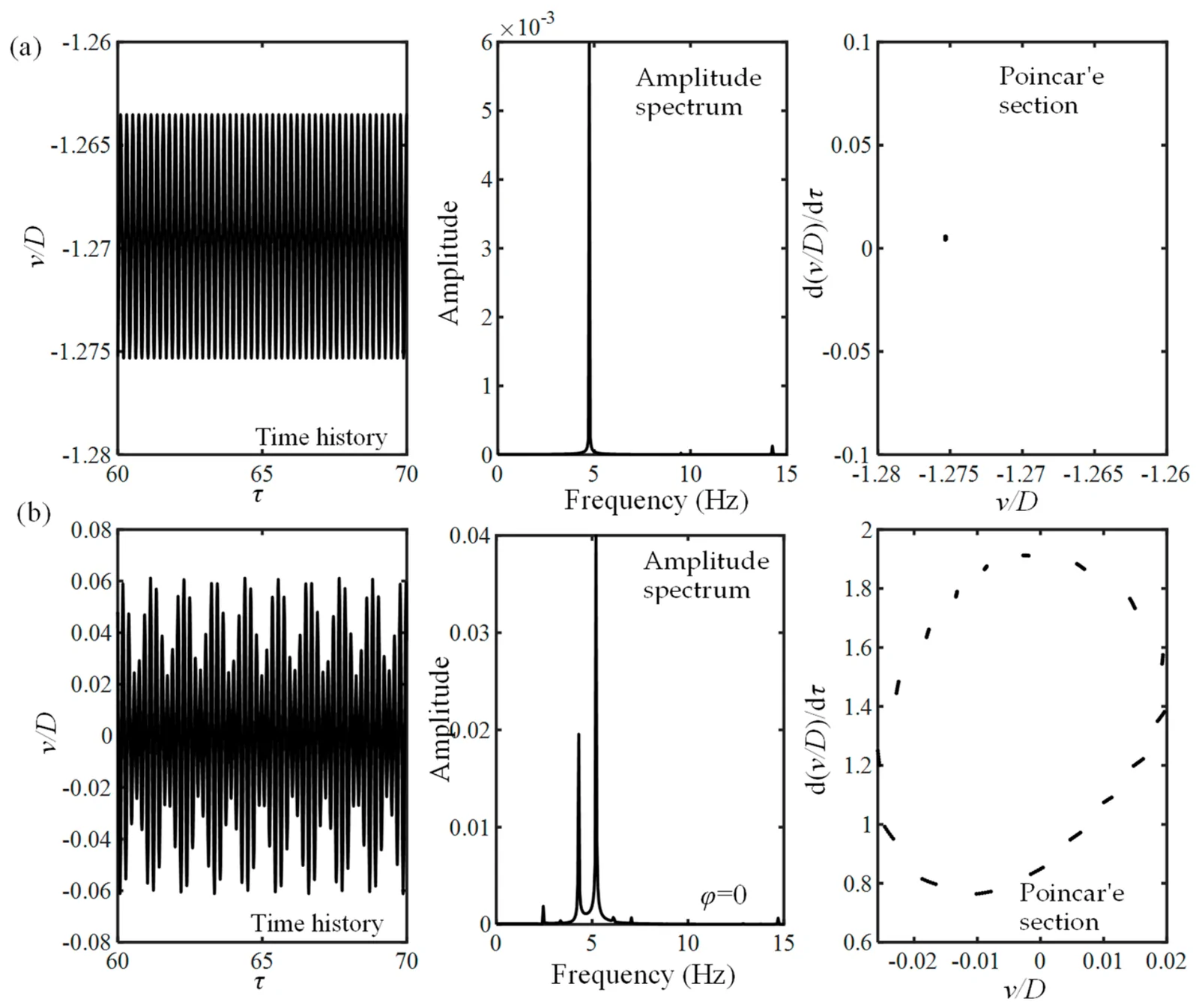
Comparison of the dynamic behaviors of the pipe under the conditions of *γ* = 10, *V* = 20 m/s, *ξ* = 0.75 and *U_e_* = 0.6769 m/s. (**a**) Considering gravity effect and (**b**) without considering gravity effect.

Figure 17 presents the bifurcation diagrams at different positions along the pipe under the gravity effect for increasing reduced velocities. In Figure 17, the discrete points represent the extrema of the time–history curve of the pipe at *ξ* = 0.5 and 0.75 over a period of time. The distribution of points in the bifurcation diagram provides a preliminary indication of the dynamic characteristics of the structure. A concentrated distribution suggests more regular motion, while a scattered distribution may indicate complex dynamic behavior. Figure 17 shows that gravity affects the vibration characteristics of the pipe. Compared with the case without gravity, the gravity-induced initial deformation changes the periodicity of the pipe motion. For example, when the external flow velocity *U_e_* approaches 0.6769 m/s, corresponding to approximately *U_r_* = 2.6 in Figure 17a and *U_r_* = 9.5 in Figure 17b, the straight pipe shows features of quasi-periodic motion at *ξ* = 0.75, whereas the pipe with gravity effect shows a more periodic response. To further support this observation, Figure 18 is presented. Figure 18 shows the time histories, amplitude spectra, and Poincaré sections of the pipe with gravity effect (*φ* ≠ 0) and the straight pipe (*φ* = 0). It can be observed that, under the same external flow velocity, the gravity-induced initial deformation reduces the dominant vibration frequency and changes the amplitude spectrum from multiple peaks to a single dominant peak. Meanwhile, the Poincaré section changes from a circular trajectory to a single point, indicating that the vibration response transitions from quasi-periodic motion to periodic motion.

### 4.3. Effect of Internal Flow Velocity

**Figure 19:**
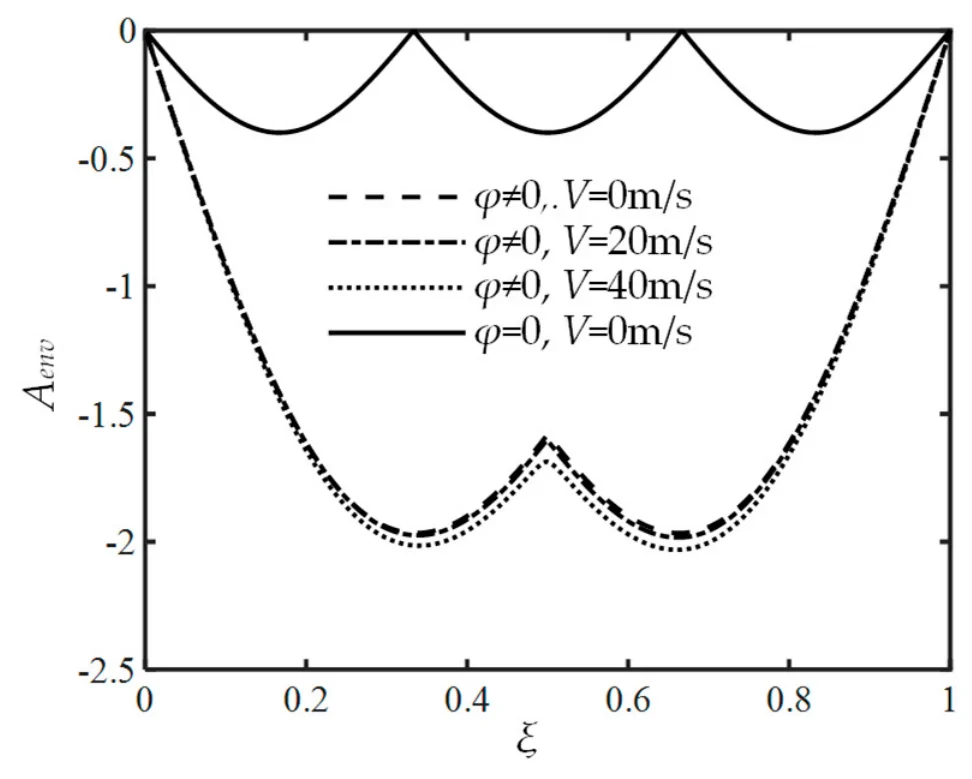
Maximum VIV Responses of the pipe with different internal flow velocities when *U_e_* = 2 m/s and *γ* = 10.

**Figure 20:**
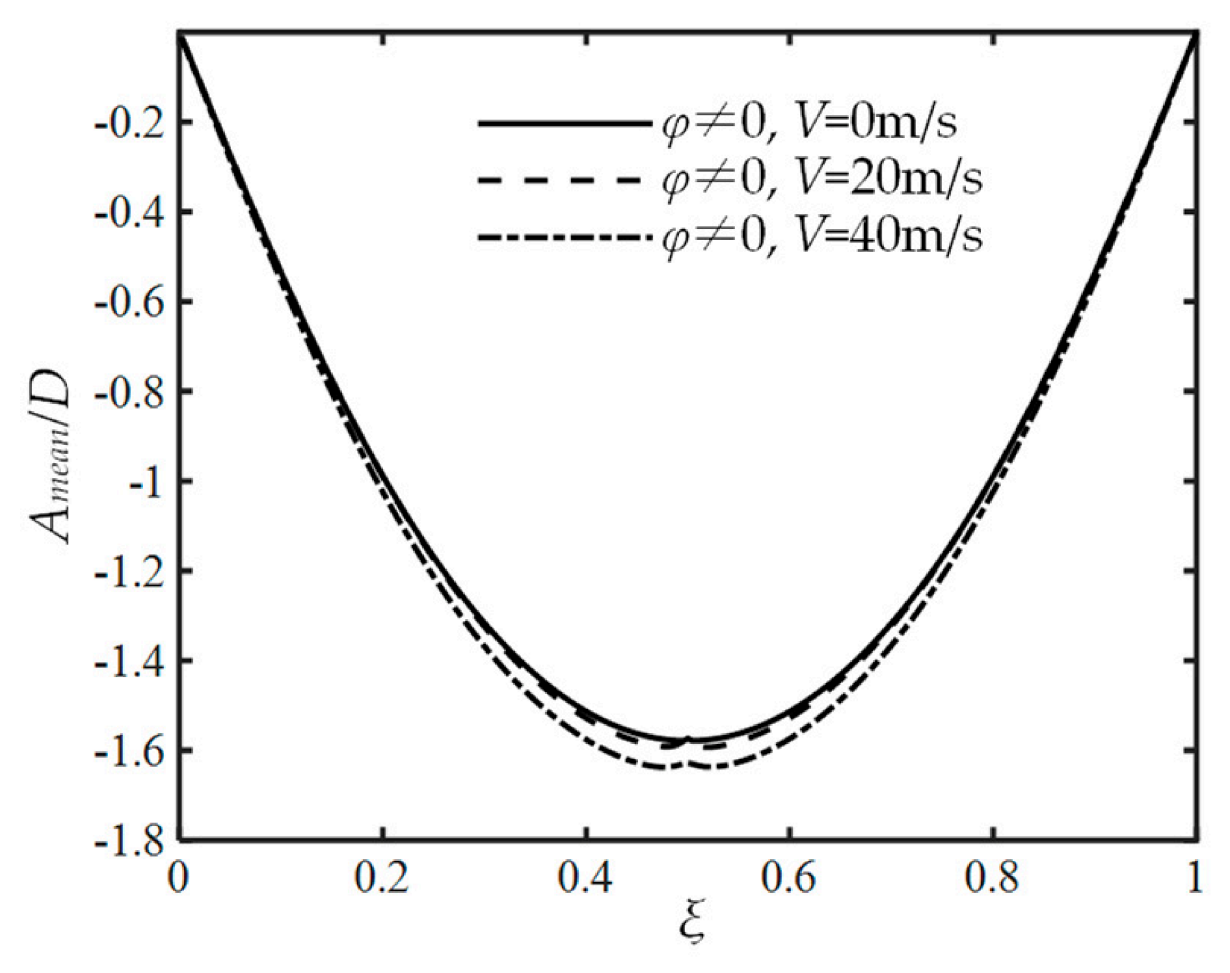
Average response amplitudes of the slightly curved pipe with different internal flow velocities when *U_e_* = 2 m/s and *γ* = 10.

This subsection investigates the effects of gravity on the pipe’s VIV amplitudes under different internal flow velocities. Figure 19 shows the maximum VIV responses of the pipe with and without the gravity effect. As shown in Figure 19, the maximum response amplitude of the pipe considering the gravity effect changes only slightly with increasing internal flow velocity. The averaged response amplitude along the pipe is further calculated and presented in Figure 20. The results show that the averaged response amplitude increases with increasing internal flow velocity. It is clearly shown in Figure 19 that, under the same operating conditions, the gravity effect reduces the response mode of the structure. Specifically, the structural response changes from the third-order mode to the second-order mode.

**Figure 21:**
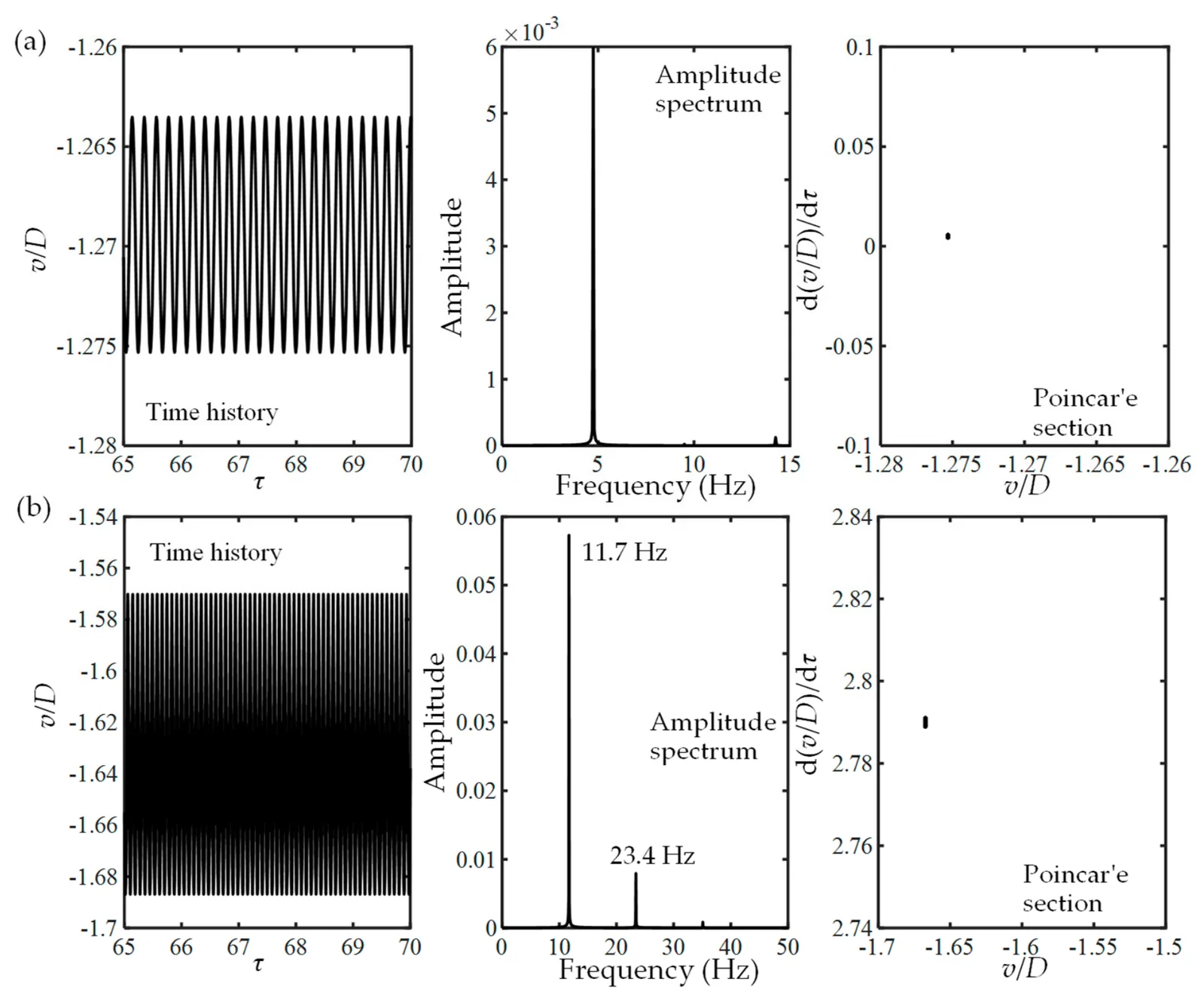
Comparison of the dynamic behaviors of the slightly curved pipe under *γ* = 10, *ξ* = 0.5 and *U_e_* = 2 m/s. (**a**) V = 20 m/s and (**b**) V = 40 m/s.

The time histories, amplitude spectra, and Poincaré sections at the midpoint of the pipe under different internal flow velocities are presented in Figure 21. It can be observed that increasing the internal flow velocity increases the dominant vibration frequency. For *V* = 20 m/s, the response is dominated by a single frequency component, and the Poincaré section contains a single point, indicating a periodic motion. When the internal flow velocity increases to *V* = 40 m/s, the dominant frequency increases from 4.75 Hz to 11.7 Hz, accompanied by a second harmonic component at 23.4 Hz. The second harmonic frequency is exactly twice the fundamental frequency, suggesting nonlinear characteristics in the structural response. However, the Poincaré section remains concentrated at a single point, indicating that the system still exhibits stable periodic oscillations.

### 4.4. Effect of Tension

During the operation of subsea pipelines, the axial tension may vary due to changes in internal pressure, internal flow velocity, and temperature of the transported fluids. These variations can modify the structural stiffness and consequently affect the dynamic behavior of the pipe. In addition, transient operating conditions, such as flow-rate fluctuations and pressure oscillations, may introduce time-dependent tension variations. Therefore, the influence of tension variation on the VIV behavior of pipes has received considerable attention [39,46]. This subsection investigates the effects of axial tension variation on the dynamic responses of slightly curved horizontal pipes under the gravity effect.

The present study focuses on the mechanism of tension variation effects on the VIV behaviors. Since the proposed model considers the pipe with slight initial curvature induced by gravity, the axial tension cannot be too small. A low axial tension may result in excessive initial deformation, which violates the assumption of slight initial curvature. Therefore, the nondimensional tension parameter is selected in the range of 10–30, corresponding to an axial tension range of 56.6–169 kN.

**Figure 22:**
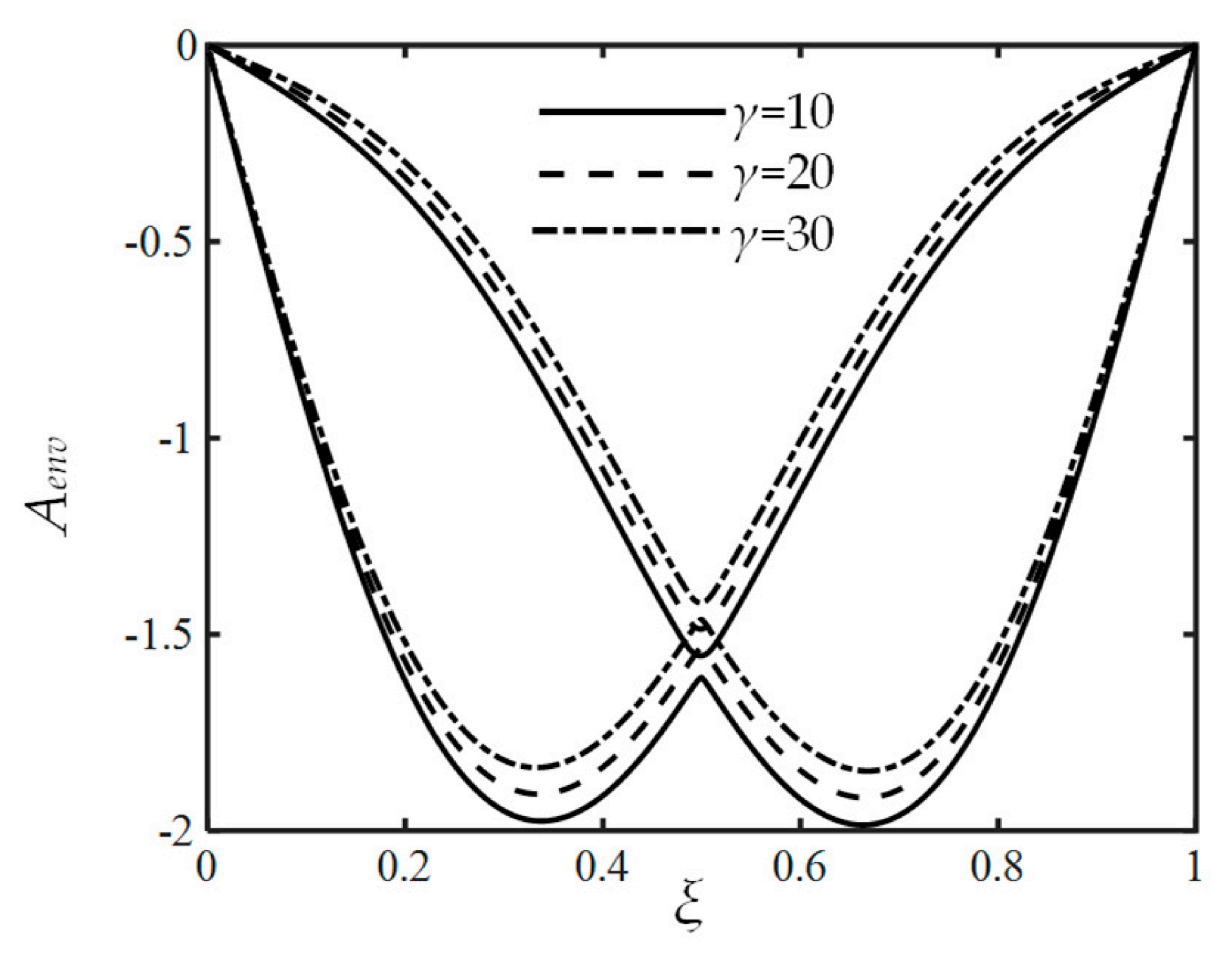
Response envelopes of the slightly curved pipes under different tension when *U_e_* = 2 m/s and *V* = 20 m/s.

**Figure 23:**
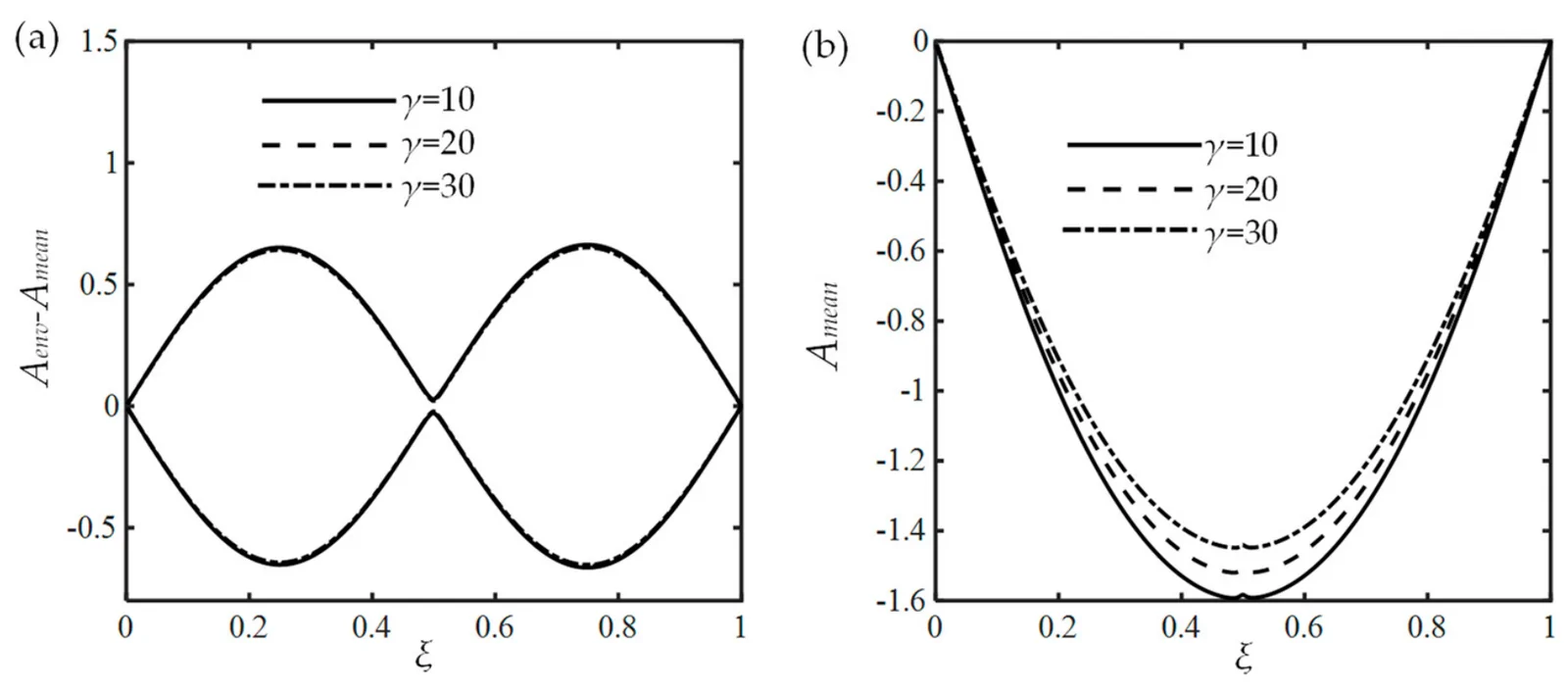
VIV responses of the slightly curved pipes under the influence of gravity and tension, with *U_e_* = 2 m/s and *V* = 20 m/s. (**a**) Response envelopes after removing the mean displacement and (**b**) average response amplitudes.

Figure 22 shows the response envelopes of the pipe considering the gravity effect, and Figure 23 presents the corresponding oscillatory displacement and averaged response displacement. As shown in Figure 22, the maximum response amplitude of the pipe decreases with increasing tension when the gravity effect is considered. The maximum response amplitude consists of the averaged displacement and the oscillatory displacement. Figure 23 shows that the decrease in averaged displacement becomes more significant as the tension increases. Therefore, the reduction in averaged displacement dominates the variation in the maximum response amplitude.

To characterize the tension variation, the dimensionless tension is further represented as

(31) $$\gamma \left(\tau \right)=\gamma_{0}+\gamma_{1}\cos\left(\Omega_{t}\tau \right),$$

where *γ*_0_ denotes the mean pretension, which is set to 10 in the following calculations, *γ*_1_ represents the amplitude of the tension variation, and $\Omega_{t}=\omega_{t}L^{2}\sqrt{\left(m_{a}+m_{f}+m_{p}\right)/EI}$ is the dimensionless form of the tension variation frequency *ω_t_*.

When the tension varies with time, the gravity-induced static configuration of the pipe also changes accordingly. In principle, the instantaneous static configuration *φ*(*γ*(*τ*)) should be updated at each time step. However, this procedure requires repeatedly solving the nonlinear equilibrium equation during the dynamic simulation, resulting in a significantly increased computational cost. Therefore, the static configuration corresponding to the mean tension *φ*(*γ*_0_) is retained throughout the calculation. To ensure the validity of this approximation, only high-frequency tension variations are considered in the present study. The frequency ratio is selected within the range of 15 < *ω_t_*/*ω*_1_ < 25. Under this condition, the static configuration cannot respond rapidly enough to follow the tension variation, and the frozen-equilibrium approximation is therefore reasonable. In addition, the tension variation amplitude is assumed to be small. The maximum value of *γ*_1_ is set to 3. The influence of the tension variation amplitude on the gravity-induced static configuration is quantified by defining

(32) $$e_{upp}=\frac{{\left\|\varphi \left(\gamma_{0}+3\right)-\varphi \left(\gamma_{0}\right)\right\|}_{L^{2}}}{{\left\|\varphi \left(\gamma_{0}\right)\right\|}_{L^{2}}},\;e_{bot}=\frac{{\left\|\varphi \left(\gamma_{0}-3\right)-\varphi \left(\gamma_{0}\right)\right\|}_{L^{2}}}{{\left\|\varphi \left(\gamma_{0}\right)\right\|}_{L^{2}}}$$

where *φ*(*γ*_0_ + 3), *φ*(*γ*_0_ − 3) and *φ*(*γ*_0_) represent the gravity-induced static configurations corresponding to tensions of *γ*_0_ + 3, *γ*_0_ + 3 and *γ*_0_, respectively. Based on Equation (26), the calculated values are *e_upp_* = 1.3192% and *e_bot_* = 1.3219%. These small deviations indicate that the variation of the static configuration caused by the time-dependent tension is negligible. Therefore, using the frozen static configuration corresponding to the mean pretension is justified in the present analysis. During the calculations, the external flow velocity is taken as *U_r_* = 4. At this flow velocity, the structure undergoes first-order lock-in resonance, as shown in Figure 15.

**Figure 24:**
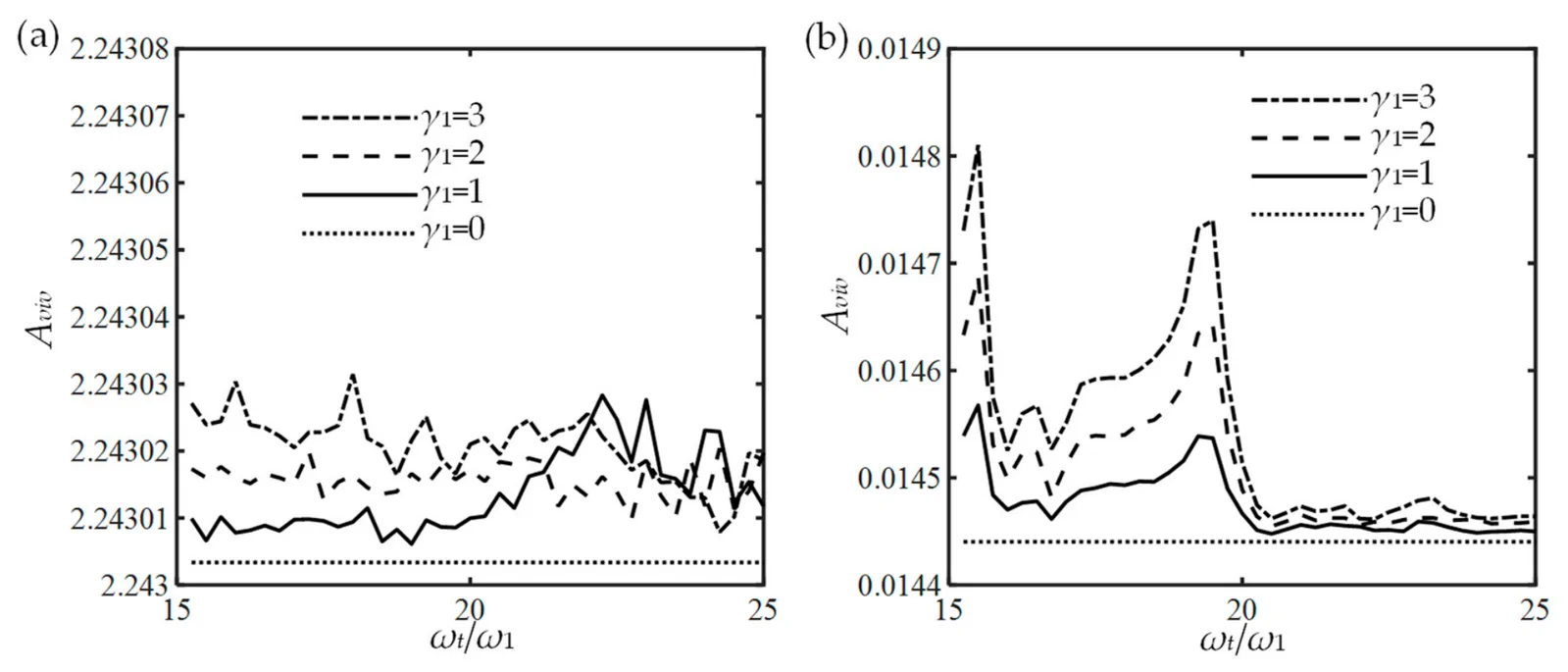
Maximum response amplitudes of the slightly curved pipes with varying tension when *ξ* = 0.5, *U_e_* = 1.0464 m/s, *V* = 20 m/s, and *γ*_0_ = 10. (**a**) Considering gravity effects and (**b**) without considering gravity effects.

Figure 24 shows the maximum VIV response amplitudes of the pipe under different tension variation amplitudes and frequencies. For comparison, the case of *γ*_1_ = 0, corresponding to the pipe subjected to constant tension, is also included in Figure 24. As shown in Figure 24a, the response amplitude remains almost unchanged for different values of *γ*_1_, especially when the tension variation frequency is relatively high. This indicates that the high-frequency tension fluctuation has a negligible influence on the maximum structural response. Compared with the constant-tension case (*γ*_1_ = 0), the introduction of tension variation slightly increases the VIV response amplitude. This phenomenon is observed for both the straight pipe and the gravity-induced slightly curved pipe, indicating that the tension fluctuation can enhance the VIV response regardless of the initial configuration.

## 5. Conclusions

The VIV prediction model of fluid-conveying pipes with gravity-induced initial slight curvature is suggested based on the conventional Euler–Bernoulli beam theory. The differences in the VIV behaviors with and without consideration of gravity-induced initial slight curvature are analyzed under variations in internal flow, external flow, and tension. The structural model is established based on Hamilton’s principle. The excitation of the external flow is modeled by the van der Pol equation with the acceleration scheme to interact with the structural motion governing equation. The dynamic equations are discretized using the Galerkin method, and the derived equations are solved using the Runge–Kutta method. The main conclusions are drawn as follows:

Gravity induces a static deformation in the pipe structure. This deformation is not a prescribed first-order sinusoidal shape and can affect the VIV response of the pipe. The static deformation decreases with increasing axial tension, while it increases with increasing internal flow velocity.

Under the same external flow velocity, the gravity-induced initial deformation reduces the dominant vibration frequency and causes the vibration response to transition from quasi-periodic to periodic motion.

The proposed model provides a reduced-order framework for investigating the role of gravity-induced initial curvature in the VIV behavior of subsea free-span pipelines. The results show that gravity-induced curvature can modify the natural frequencies and nonlinear dynamic responses of the pipe. However, due to the simplified assumptions adopted in the model, the results should be regarded as qualitative insights rather than direct engineering predictions. Future studies should consider more realistic boundary conditions, pipe–soil interactions, nonuniform currents, three-dimensional vibration responses, and experimental validation.

## Figures and Tables

**Table 1:** Description of dimensionless parameters.

Parameters	Descriptions	Ranges
$\xi =\frac{x}{L}$	Nondimensional coordinate	0–1
$\bar{v}=\frac{v}{D}$	Nondimensional displacement	\
$\tau =\sqrt{\frac{EI}{\left(m_{a}+m_{f}+m_{p}\right)}}\frac{t}{L^{2}}$	Nondimensional time	\
$\chi =\frac{AD^{2}}{I}$	Ratio of axial stiffness to bending stiffness	\
$\beta =\frac{m_{f}}{m_{a}+m_{f}+m_{p}}$	Mass ratio of internal fluid to total mass	\
$\Omega_{s}=\omega_{s}\sqrt{\frac{\left(m_{a}+m_{f}+m_{p}\right)}{EI}}L^{2}$	Nondimensional vortex shedding frequency	0–87.8
$\sigma_{f}=\frac{C_{D}\rho_{0}DU_{e}L^{2}}{2\sqrt{EI\left(m_{p}+m_{f}+m_{a}\right)}}$	Nondimensional hydrodynamic damping parameter	\
$\sigma_{s}=\frac{cL^{2}}{\sqrt{EI\left(m_{p}+m_{f}+m_{a}\right)}}$	Nondimensional structural damping parameter	\
$\gamma =\frac{L^{2}T_{e}}{EI}$	Nondimensional axial tension	0–30
$u_{i}=\sqrt{\frac{m_{f}}{EI}}LV$	Nondimensional internal flow velocity	<4.8
$\Gamma =\frac{\left(m_{f}+m_{p}-\frac{\rho_{0}\pi D^{2}}{4}\right)gL^{4}}{EID}$	Nondimensional gravity parameter	\
$\alpha =\frac{C_{L0}\rho_{0}U_{e}^{2}L^{4}}{4EI}$	Nondimensional lift parameter	\

**Table 2:** Parameters of the pipe model [55].

Parameters	Descriptions	Values
*L*	Length (m)	150
*D*	Outer diameter (m)	0.25
*D_i_*	Inner diameter (m)	0.125
*E*	Elastic modulus (GPa)	210
*ρ_p_*	Density of pipe (kg/m^3^)	7850
*ρ*_0_	Density of external fluid (kg/m^3^)	1020
*ρ_i_*	Density of internal fluid (kg/m^3^)	870

**Table 3:** Parameters of the pipe model [63].

Parameters	Descriptions	Values
*L*	Length (m)	38
*D*	Outer diameter (m)	0.027
*D_i_*	Inner diameter (m)	0.021
*E*	Elastic modulus (GPa)	36.2
*m_p_*	Structure mass (kg/m^3^)	0.939
*ρ*_0_	Density of external fluid (kg/m^3^)	1020
*U_e_*	External flow velocity (m/s)	0.4
*T_e_*	Tension (kN)	6

**Table 4:** Temporal convergence analysis of the numerical method.

Δ*τ*	Error *e*_Δ*τ*_	Order *p*
0.002	8.6544 × 10^−5^	1.9994
0.001	2.1637 × 10^−5^	1.9999
0.0005	5.4054 × 10^−6^	2.001
0.00025	1.3514 × 10^−6^	2

**Table 5:** Structural and material parameters of the pipe [67].

Parameters	Descriptions	Values
*L*	Length (m)	50
*D*	Outer diameter (m)	0.24
*D_i_*	Inner diameter (m)	0.21
*E*	Elastic modulus (GPa)	210
*ρ_p_*	Density of pipe (kg/m^3^)	7800
*ρ*_0_	Density of external fluid (kg/m^3^)	1025
*ρ_i_*	Density of internal fluid (kg/m^3^)	870

**Table 6:** Modal coefficients and relative contributions of the static deformed configuration of the pipe under gravity.

Mode	${\bar{\bar{v}}}_{i}\left(\tau \right)$	$\frac{{\left|{\bar{\bar{v}}}_{i}\left(\tau \right)\right|}^{2}}{\sum_{i}{\left|{\bar{\bar{v}}}_{i}\left(\tau \right)\right|}^{2}}$
1	−1.24783	99.9616%
2	0	0%
3	−0.02431	0.0379%
4	0	0%
5	−0.00269	0.0005%
6	0	0%

## Data Availability

The original contributions presented in this study are included in the article. Further inquiries can be directed to the corresponding author.
